# C-reactive protein reduction post treatment is associated with improved survival in atezolizumab (anti-PD-L1) treated non-small cell lung cancer patients

**DOI:** 10.1371/journal.pone.0246486

**Published:** 2021-02-03

**Authors:** Namrata S. Patil, Wei Zou, Simonetta Mocci, Alan Sandler, Marcus Ballinger, Susan Flynn, Marcin Kowanetz, Priti S. Hegde

**Affiliations:** Genentech, S. San Francisco, California, United States of America; Goethe University Hospital Frankfurt, GERMANY

## Abstract

**Purpose:**

Overall survival (OS) is the most significant endpoint for evaluation of treatment benefit with checkpoint inhibitors (CPI) in cancer. We evaluated serum C-reactive protein (CRP) in non-small cell lung cancer (NSCLC) trials with atezolizumab (anti-PD-L1) as an early OS surrogate.

**Methods:**

Serum from patients enrolled in randomized Phase II (n = 240) and Phase III (n = 701) trials of NSCLC patients (POPLAR, OAK) who progressed on prior-platinum chemotherapy, were analyzed for CRP levels over time. Patients were grouped by changes in CRP levels post-treatment as either increased (≥ 1.5 fold), decreased (≤ 1.5 fold) or unchanged (within +1.5 fold) relative to pre-treatment levels to assess association with progression free survival (PFS) and OS.

**Results:**

Decrease in serum CRP levels at 6 weeks relative to pre-treatment were observed in patients with RECIST1.1 based complete or partial responses (CR/PR) to atezolizumab whereas patients with disease progression (PD) demonstrated an increase in CRP levels in the Phase II POPLAR study, and confirmed in the Phase III OAK study. Decrease in serum CRP as early as six weeks post treatment predicted improved PFS and OS, even in patients who were determined as stable disease (SD) in their first scan. This effect was not observed in the chemotherapy arms.

**Conclusion:**

Modulation of serum CRP correlates with clinical outcome post-atezolizumab treatment. This routine lab test may provide utility in informing OS signals as early as 6 weeks post-initiation of therapy with CPIs in NSCLC.

## Introduction

The concept of harnessing one’s immune system to recognize and kill tumor cells has proven to be transformative for therapeutic intervention in cancer. The most promising feature of this approach is the long-lasting durable benefit observed in patients across a wide range of cancer types [1]. Indeed, objective response rates (ORR) and progression free survival (PFS) underestimate the overall survival (OS) benefit achieved by cancer immunotherapeutic agents [2]. Thus, overall survival has now become the most relevant and widely studied primary endpoint in clinical trials investigating programmed death-1 (PD-1) and programmed death-ligand 1 (PD-L1) targeted agents.

In clinical development, interrogation of OS as an endpoint can take several years to investigate. Furthermore, OS can be confounded by patients on the control arm either crossing-over or receiving in-class agents in later lines of treatment in randomized clinical trials. This can result in significant underestimation of the true OS benefit when evaluating hazard ratios between the treatment and control arms [3]. Given that there are over 1500 clinical trials underway with over 900 molecules in combination with checkpoint inhibitors, there is an urgent need to identify early surrogates of OS benefit that can enable early and informed decisions in these trials particularly as it pertains to prioritization of combinations or agents for further drug development.

Non-invasive biomarkers, such as the assessment of minimal residual disease (MRD) in hematologic malignancies, are gaining significant traction as potential regulatory endpoints for OS in clinical trials [4]. In solid tumors, the presence of peripheral tumor burden markers has been used as potential surrogates particularly in diseases such as Ovarian cancer (CA-125) [5], prostate cancer (PSA) [6] and pancreatic cancer (CA19-9) [7]. In addition, systemic inflammatory response markers such as C-reactive protein (CRP) have been associated with poor prognosis in patients with many types of cancer [8], including non-small cell lung cancer [9], one of the leading causes of cancer-related deaths worldwide.

In this study, we explored the utility of changes in serum C-reactive protein (CRP) levels as a potential on-treatment surrogate of OS in two randomized clinical trials of atezolizumab (anti-PD-L1) monotherapy in second-line non-small cell lung cancer (NSCLC) [10, 11]. The findings from the randomized Phase II study POPLAR were independently validated in the larger Phase III study OAK, thus demonstrating the robustness of a simple and routine lab-based test in predicting survival with monotherapy checkpoint inhibition as early as 6 weeks after initiation of therapy.

## Materials and methods

### Patient population

This study was performed using serum samples from the open-label, randomized Phase II POPLAR (NCT01903993, n = 287) and Phase III OAK trials (NCT02008227 primary analysis n = 850) that evaluated atezolizumab vs docetaxel in patients with NSCLC who progressed on post-platinum chemotherapy [10, 11]. Patients in both trials received either 1200 mg atezolizumab IV every 3 weeks (q3w) until disease progression (PD) or loss of clinical benefit, or 75 mg/m^2^ docetaxel IV q3w until PD. Both studies demonstrated significant improvement in OS with atezolizumab versus docetaxel, regardless of PD-L1 expression [11]. All procedures were in accordance with the ethical standards of the responsible committees and IRBs on human experimentation. All IRBs approved the study prior to data collection, and all patients gave written informed consent to participate in these studies. Patients were recruited between Aug 2013, and March 2014 for the POPLAR study and between March 2014, and April 2015 in the OAK study.

### CRP measurement

Peripheral venous blood was obtained under sterile conditions from patients prior to treatment (on cycle 1, day 1, predose) and every 3 weeks thereafter. Serum was separated by centrifuging for 10 min at 1000 g and 4°C in vacuum gel tubes, and then kept at –80°C until the time of assay. CRP was analyzed by routine clinical laboratory test protocols utilizing an automated chemical analyzer using the high sensitivity Siemens HS-CRP assay for this study, though in practice any validated CRP assay can be used since a time-dependent relative ratio method is used, as described below.

### Data analysis

Baseline and six weeks post-treatment serum CRP data was available from n = 190 (POPLAR) and n = 558 (OAK) patients. Patients who did not have week six CRP data or CT- scan data were excluded from the biomarker evaluable population (BEP). Patient demographics between primary-analysis ITT and BEP populations in the POPLAR and OAK studies were similar across both treatment arms (OAK study BEP was N = 256 for Docetaxel and N = 302 for atezolizumab; see S1 and S2 Tables). From a molecular epidemiologic perspective in healthy donors, reported variability in CRP between two serial values is considered to be ~ 120% [12]. Thus, a conservative estimate of 150% change in CRP from baseline measurements was considered to be a meaningful difference for the purpose of this study. This was significantly above the reported assay variability of 6.8% [13]. CRP was analyzed as a continuous, categorical, and time-dependent variable. CRP fold changes (FC) at 6 weeks (cycle 3 day 1 predose or C3D1) were categorized as increase if they were greater than 1.5 fold relative to baseline or cycle 1 day 1 predose or C1D1 (ratio ≥ 1.5), as decrease if less than 1.5 fold relative to baseline (ratio ≤ 0.67) or unchanged if in between these limits (ratio 0.67 to <1.5). Patients were grouped by CRP fold changes and/ or by investigator assessed response at 6 weeks according to the Response Evaluation Criteria in Solid Tumors (RECIST) v1.1 criteria. PFS and OS were re-baselined to their CRP collection day for their week 6 sample; patients censored or with an event before this time were excluded from this analysis. Hazard ratios (HR) comparing atezolizumab and docetaxel arm in CRP bins and/or response bins were estimated from cox proportional models. Time-dependent receiver operating characteristic (ROC) curves were generated from censored survival data using the Nearest Neighbor Estimation (NNE) method using R package survival ROC version 1.0.3. Confidence intervals for the area under curve (AUC) were estimated from bootstrap.

## Results

### POPLAR demonstrates proof of principle for time dependent CRP modulation as a predictor of clinical response

**Fig 1:**
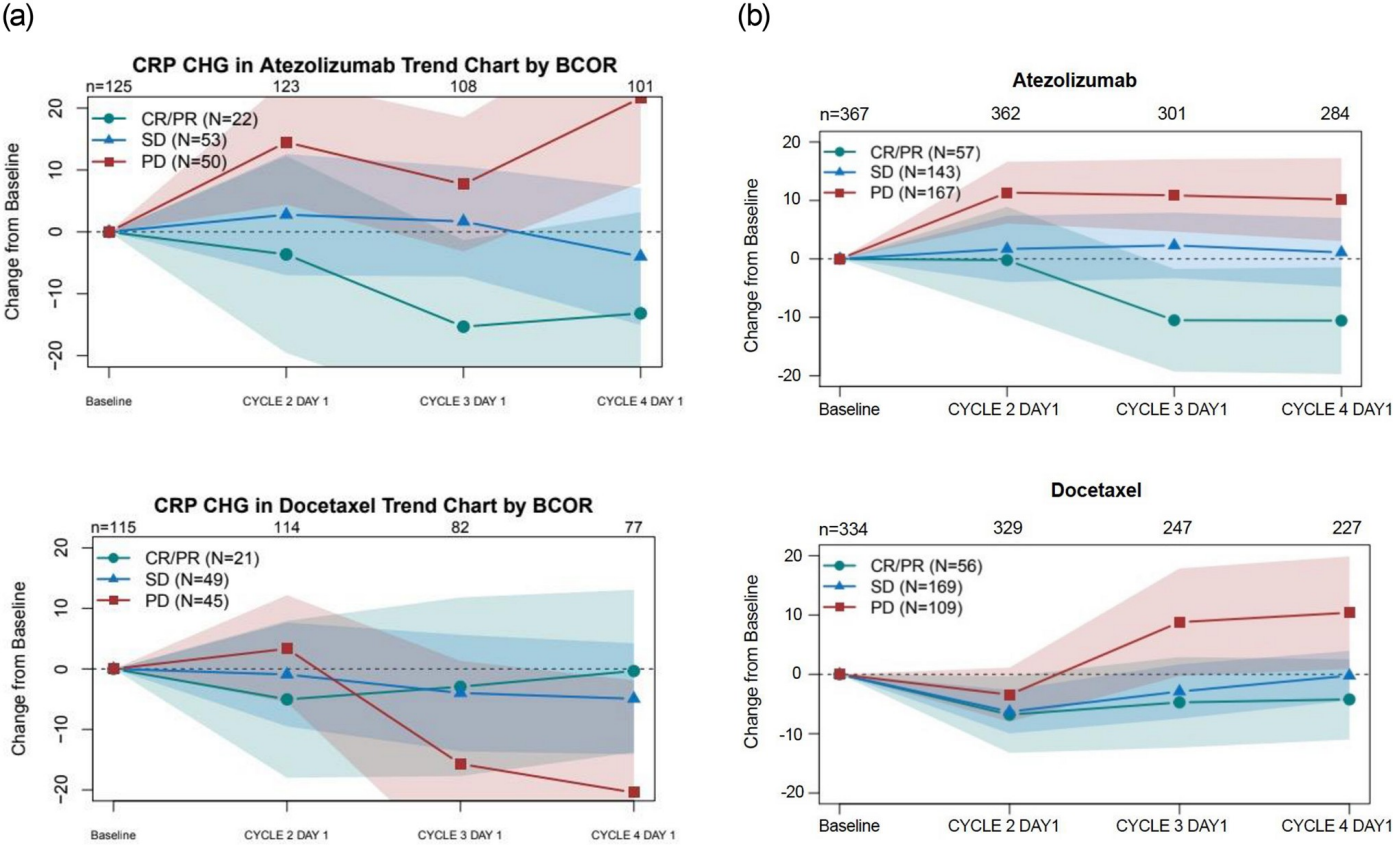
Modulation of CRP relative to pre-treatment is associated with overall response post atezolizumab treatment but not with docetaxel treatment in the POPLAR and OAK studies. **A.** POPLAR Studies **B.** OAK Studies. Trend plots of CRP change from baselines of patients in different response categories in both the POPLAR and OAK study using simple means for each visit separately. Shaded regions indicate point-wise 95% confidence intervals. n: number of patients with a change from baseline value at a particular visit, excluding patients with missing or not evaluable best confirmed overall response. N: number of patients at baseline in each best confirmed overall response category.

To examine the relationship between clinical outcomes and CRP changes, we evaluated all available baseline (C1D1 pre-dose sample) and post-treatment serum CRP data available in the POPLAR study. The samples analyzed were taken every three weeks post-dosing. In the majority of patients treated with atezolizumab that were designated as PR by best-confirmed overall response (BCOR), mean CRP levels post-treatment decreased relative to pre-treatment in the atezolizumab treated arm (Fig 1a), whereas patients designated as PD showed a mean increase in CRP levels relative to pre-treatment. This association between changes in serum CRP and RECIST response was not observed in patients treated with chemotherapy.

### OAK validates decrease in serum CRP as a predictor of clinical response

To confirm this association of CRP changes with response, we next sought to independently validate these findings by analyzing CRP levels in the pivotal Phase III OAK study. Similar to the POPLAR study results, mean CRP changes post-treatment relative to baseline were associated with BCOR in atezolizumab-treated patients; decreasing in patients with a response to therapy and increasing in patients with progressive disease (Fig 1b). The chemotherapy arm showed a mean increase in serial CRP levels in progressing patients.

**Fig 2:**
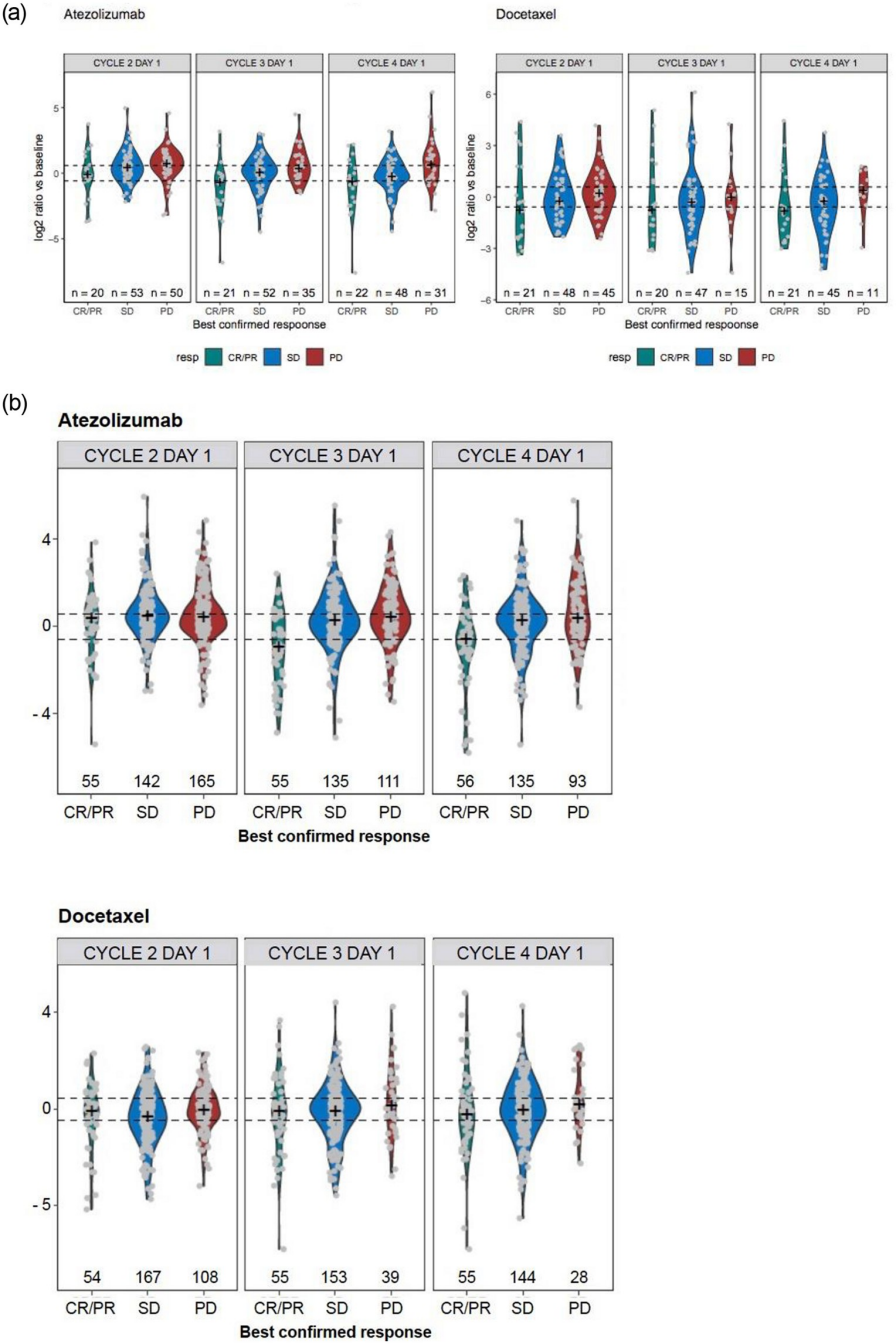
CRP ratios relative to pre-treatment is associated with overall response post-atezolizumab treatment but not with docetaxel treatment in the POPLAR and OAK studies. CRP log 2 ratio relative to pre-treatment or C1D1 pre-dose for each patient for each treatment arm for the first four visits with dotted lines marking an increase (1.5 fold increase) or decreased (1.5 fold decrease) relative to pre-treatment. CR, complete response; CRP, c-reactive protein; PD, disease progression; PR, partial response; RECIST v1.1, Response Evaluation Criteria in Solid Tumors version 1.1; SD, stable disease.

To determine if CRP modulation may be associated with clinical outcome, the fold change in CRP at C2D1, C3D1 and C4D1 were plotted for each RECIST 1.1 response category or BCOR of PR, SD and PD for patients in both the POPLAR (Fig 2a) and OAK studies (Fig 2b). Despite median decreases observed in PR patients and median increases in CRP in patients with PD, a wide range of CRP modulation was observed in all three groups, including some SD and PD patients who showed a significant reduction in circulating CRP. To investigate a patient level PFS and OS association with CRP changes, patients were classified as (1) CRP increase (≥1.5 fold), (2) CRP decrease (≤1.5 fold) or (3) no change (±1.5 fold) relative to baseline (Table 1). Here we present the analyses from the OAK study since it is a significantly larger study. Clinicopathological and demographic variables for each of these CRP groups (increase, decrease, no change) at 6 weeks is shown in Tables 2 and 3. ECOG status appeared to be evenly distributed in these groups, suggesting CRP decrease at six weeks did not correlate with known risk-factors associated with inherently healthier patients.

**Table 1:** PFS and OS benefit based on CRP changes at 6 weeks in OAK.

**CRP change @ 6 weeks**	**Prevalence in atezolizumab arm N (%)**	**Prevalence in docetaxel arm N (%)**	**Median PFS atezolizumab**	**Median PFS docetaxel**	**HR (rel. to docetaxel) (95% CI)**	**P Value**
Increase (≥1.5 fold)	83 (34%)	73 (29%)	2.83	4.07	0.96 (0.69–1.34)	0.81
No Change (±1.5 fold)	93 (38%)	92 (37%)	3.55	4.17	0.85 (0.62–1.15)	0.28
Decrease (≤1.5 fold)	68 (28%)	83 (33%)	8.97	4.11	0.48 (0.33–0.69)	9.5E-5
**CRP change @ 6 weeks**	**Prevalence in atezolizumab arm N (%)**	**Prevalence in docetaxel arm N (%)**	**Median OS atezolizumab**	**Median OS docetaxel**	**HR (rel. to docetaxel) (95% CI)**	**P Value**
Increase (≥1.5 fold)	113 (37%)	76 (30%)	12.22	15.44	1.25 (0.86–1.81)	0.25
No Change (±1.5 fold)	114 (38%)	93 (36%)	16.66	10.55	0.61 (0.43–0.86)	0.0049
Decrease (≤1.5 fold)	75 (25%)	87 (34%)	21.75	10.58	0.49 (0.32–0.75)	0.0011

PFS: progression free survival; OS: overall survival; CRP: C-reactive protein; HR: hazard ratio. Cox models were fit among patients who were at risk for PFS/OS at 6 weeks.

**Table 2:** Patient demographics and clinical characteristics in the three CRP fold change buckets for atezolizumab in OAK.

	No Change	Decrease	Increase
	[-1.5,1.5]	< -1.5	>=1.5
**AGE**			
N	114	75	113
Mean	63.7	62.44	62.44
Min-Max	35…82	44…81	42…82
blSLD			
N	113	75	113
Mean	72.06	68.79	78.79
Min-Max	10…203	15…261	14…316
**SEX**			
Total	114	75	113
F	41 (35.96%)	26 (34.67%)	57 (50.44%)
M	73 (64.04%)	49 (65.33%)	56 (49.56%)
**HISTOLOGY**			
Total	114	75	113
NON-SQUAMOUS	83 (72.81%)	55 (73.33%)	90 (79.65%)
SQUAMOUS	31 (27.19%)	20 (26.67%)	23 (20.35%)
**ECOGGR**			
Total	114	75	113
0	48 (42.11%)	31 (41.33%)	47 (41.59%)
1	66 (57.89%)	44 (58.67%)	66 (58.41%)
**SMOKING STATUS**			
Total	114	75	113
CURRENT	13 (11.4%)	17 (22.67%)	15 (13.27%)
NEVER	21 (18.42%)	11 (14.67%)	27 (23.89%)
PREVIOUS	80 (70.18%)	47 (62.67%)	71 (62.83%)
**LIVER**			
Total	114	75	113
N	101 (88.6%)	68 (90.67%)	92 (81.42%)
Y	13 (11.4%)	7 (9.33%)	21 (18.58%)
**ICLEVEL**			
Total	113	75	112
0	46 (40.71%)	37 (49.33%)	62 (55.36%)
1	53 (46.9%)	24 (32%)	40 (35.71%)
2	7 (6.19%)	12 (16%)	4 (3.57%)
3	7 (6.19%)	2 (2.67%)	6 (5.36%)
**TCLEVEL**			
Total	113	75	112
0	79 (69.91%)	43 (57.33%)	90 (80.36%)
1	7 (6.19%)	4 (5.33%)	6 (5.36%)
2	16 (14.16%)	16 (21.33%)	9 (8.04%)
3	11 (9.73%)	12 (16%)	7 (6.25%)

BlSLD: baseline tumor size or sum of longest diameter; CRP: C-reactive protein; ECOG GR: ECOG status; IC Level: PD-L1 on immune cells (0:<1%; 1:1–5%; 2:>=5–10%; 2: >=10%); TC level: PD-L1 on tumor cells (0:<1%; 1:1–5%; 2:>=5–50%; 2: >=50%).

**Table 3:** Patient demographics and clinical characteristics in the three CRP fold change buckets for docetaxel in OAK.

	No Change	Decrease	Increase
	[-1.5,1.5]	< -1.5	>=1.5
**AGE**			
N	93	87	76
Mean	65.26	60.68	62.63
Min-Max	43…85	40…84	36…80
blSLD			
N	93	87	76
Mean	66.56	79.28	71.8
Min-Max	10…207	10…204	10…198
**SEX**			
Total	93	87	76
F	39 (41.94%)	33 (37.93%)	29 (38.16%)
M	54 (58.06%)	54 (62.07%)	47 (61.84%)
**HISTOLOGY**			
Total	93	87	76
NON-SQUAMOUS	68 (73.12%)	66 (75.86%)	58 (76.32%)
SQUAMOUS	25 (26.88%)	21 (24.14%)	18 (23.68%)
**ECOGGR**			
Total	93	87	76
0	37 (39.78%)	38 (43.68%)	34 (44.74%)
1	56 (60.22%)	49 (56.32%)	42 (55.26%)
**SMOKING STATUS**			
Total	93	87	76
CURRENT	19 (20.43%)	11 (12.64%)	9 (11.84%)
NEVER	18 (19.35%)	16 (18.39%)	11 (14.47%)
PREVIOUS	56 (60.22%)	60 (68.97%)	56 (73.68%)
**LIVER**			
Total	93	87	76
N	79 (84.95%)	69 (79.31%)	58 (76.32%)
Y	14 (15.05%)	18 (20.69%)	18 (23.68%)
**ICLEVEL**			
Total	92	86	76
0	52 (56.52%)	41 (47.67%)	36 (47.37%)
1	28 (30.43%)	34 (39.53%)	26 (34.21%)
2	8 (8.7%)	7 (8.14%)	11 (14.47%)
3	4 (4.35%)	4 (4.65%)	3 (3.95%)
**TCLEVEL**			
Total	92	86	76
0	64 (69.57%)	62 (72.09%)	48 (63.16%)
1	5 (5.43%)	4 (4.65%)	4 (5.26%)
2	13 (14.13%)	12 (13.95%)	16 (21.05%)
3	10 (10.87%)	8 (9.3%)	8 (10.53%)

BlSLD: baseline tumor size or sum of longest diameter; ECOG GR: ECOG status; IC Level: PD-L1 on immune cells (0:<1%; 1:1–5%; 2:>=5–10%; 2: >=10%); TC level: PD-L1 on tumor cells (0:<1%; 1:1–5%; 2:>=5–50%; 2: >=50%).

### Longitudinal CRP as a surrogate for PFS and OS in OAK

**Fig 3:**
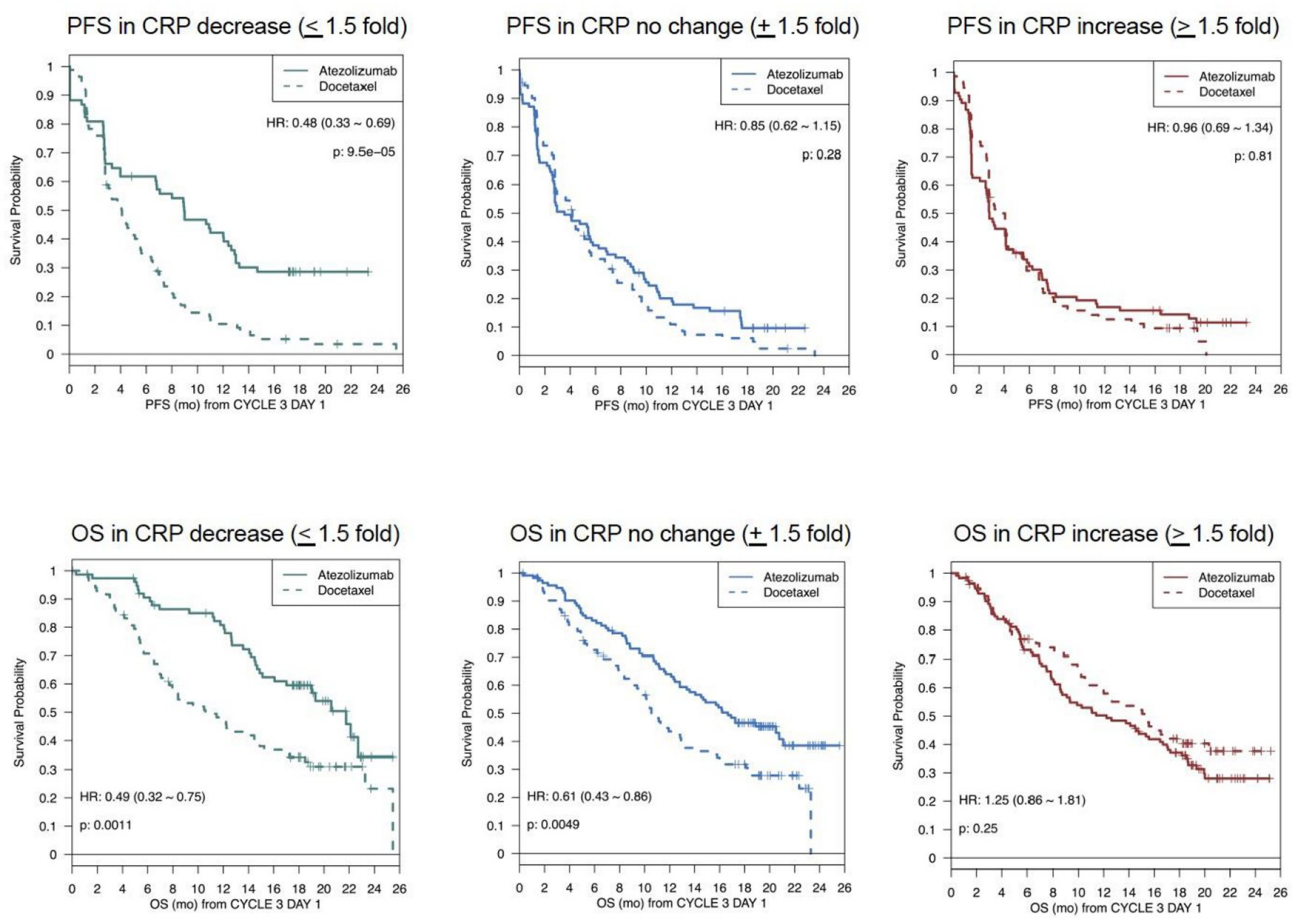
Landmark OS and PFS Kaplan-Meier curves for patients binned by CRP changes at 6 weeks. Landmark OS and PFS Kaplan-Meier curves were plotted based on patients binned by CRP fold changes at 6 weeks. **(3a**) PFS (Top) and (**3b**) OS (Bottom) were calculated from 6 weeks or cycle 3 day 1 (C3D1) post-treatment; patients censored or with an event before this time were excluded from this analysis. Patients treated with atezolizumab are in the solid lines and patients treated with docetaxel are in the broken lines. The first panel is patients who had CRP decrease (blue), the middle panel is patients who showed no change in CRP (black) and the last panel is patients who showed an increase in CRP (red). CRP, C-reactive protein; HR, hazard ratio; OS, overall survival; PFS, progression free survival.

28% of BEP treated with atezolizumab showed a decrease of CRP (≤1.5 fold) at six weeks and demonstrated PFS benefit with median PFS of 9 months vs docetaxel median PFS of 4.11 months (HR: 0.49, CI 0.34–0.7) (Fig 3a). However, patients with relatively unchanged CRP (±1.5 fold) or with CRP increase (≥1.5 fold) did not show improvement in PFS compared to docetaxel (Table 1).

In addition, change in serum CRP was significantly associated with treatment benefit as measured by OS (Fig 3b). Patients treated with atezolizumab who showed a CRP decrease at 6 weeks (~25% of BEP), with median OS of 21.7 months as compared to the median OS of 10.58 months for docetaxel (HR: 0.49, CI 0.32–0.75) (Table 1). Patients with relatively unchanged CRP (±1.5 fold, ~38% BEP) showed modest OS benefit with median OS being 16.6 months for atezolizumab as compared to median OS of 10.55 months for docetaxel (HR: 0.85, CI 0.62–1.15). However, in patients who showed a CRP increase at 6 weeks (~37% BEP), the median OS was 12.2 months for atezolizumab as compared to 15.44 months for docetaxel (HR: 1.25, CI 0.86–1.81). Notably, patients showing decrease in CRP at 6 weeks spanned a wide range of pre-treatment CRP levels and were not limited to those with higher than normal levels at baseline. Consistent with prior reports, high CRP at baseline was prognostic for poor overall survival in both treatment arms (S1 Fig).

### OS association with CRP changes in context of CT-scans at 6 weeks

To determine how serum CRP changes in comparison to CT- scan evaluations at six weeks, in terms of predicting OS, patients were analyzed based on their 6-week RECIST 1.1 CT-scan based assessment and evaluated for association with OS. In this BEP, 9% of atezolizumab treated patients were characterized as partial responders at the 6-week scan and showed significant OS improvement (median OS not reached at time of analysis) (Table 4). This is distinct from the 15% partial responders with atezolizumab in the ITT population based on best-confirmed overall response which also includes patients who responded at later scans [11].

**Table 4:** OS benefit based on ORR using RECIST 1.1 scans and CRP changes at 6 weeks in OAK.

**RECIST 1.1 at 6 weeks scan (not best confirmed overall response)**	**Prevalence in atezolizumab arm N**	**Prevalence in docetaxel arm N**	**Median OS atezolizumab**	**Median OS docetaxel**	**HR (rel. to docetaxel) (95% CI)**	**P Value**
PD	82	12	8.61	4.45	0.51 (0.27–0.98)	0.044
SD	197	216	17.28	12.19	0.67 (0.52–0.86)	0.0019
PR	21	26	not met	14.82	0.38 (0.16–0.94)	0.036
**CRP change @ 6 weeks in patients designated SD at 6 weeks scan**	**Prevalence in atezolizumab arm N**	**Prevalence in docetaxel arm N**	**Median OS atezolizumab**	**Median OS docetaxel**	**HR (rel. to docetaxel)** **(95% CI)**	**P Value**
Increase (≥1.5 fold)	69	60	15.57	15.51	1.08 (0.69–1.7)	0.73
No Change (±1.5 fold)	77	84	18.89	11.04	0.55 (0.36–0.82)	0.0032
Decrease (≤1.5 fold)	51	72	22.11	12.22	0.5 (0.3–0.82)	0.0064
**CRP change @ 6 weeks in patients designated PD at 6 weeks scan**	**Prevalence in atezolizumab arm N (%)**	**Prevalence in docetaxel arm N (%)**	**Median OS atezolizumab**	**Median OS docetaxel**	**HR (rel. to docetaxel) (95% CI)**	**P Value**
Increase (≥1.5 fold)	37	5	6.93	3.35	0.67 (0.23–1.93)	0.46
No Change (±1.5 fold)	30	5	12.32	5.39	0.55 (0.21–1.47)	0.23
Decrease (≤1.5 fold)	15	2	19.02	5.08	0.15 (0.02–0.9)	0.038

OS: overall survival; ORR: objective response rates; CRP: C-reactive protein; HR: hazard ratio; PD: disease progression; SD: stable disease; PR: partial response.

At the 6-week scan, approximately 65% of the atezolizumab treated patients were characterized as stable disease (SD) and 27% as progressive disease (PD) (Table 4). Patients designated as SD had a median OS of 17.2 months and patients designated PD had a median OS of 8.6 months. Remarkably, about one-fourth (26%) of SD patients showed CRP decrease at 6 weeks and were associated with OS benefit, with a median OS of 22.1 months with atezolizumab and 12.22 months with docetaxel. SD patients with no change in serum CRP had a median OS of 18.89 months on atezolizumab versus 11.04 months on Docetaxel. SD patients who showed CRP increase at 6 weeks had median 15.5 months survival irrespective of treatment. Similar trends were observed for PD patients, though the results were not statistically significant given the small number of patients in the distinct CRP bins (Table 4).

### Quantifying longitudinal CRP association with OS

To better quantify the predictability of dynamic CRP modulation for OS, receiver-operator curves (ROC) were plotted. The ability of CRP ratio (C3D1 vs baseline) to predict 1-year OS (from randomization) in OAK, at various cutoff values of CRP was analyzed. The AUC value for atezolizumab was 0.67 whereas the AUC value for docetaxel was 0.44, suggesting that the CRP decrease at 6 weeks is predictive with OS in the atezolizumab arm but not in the docetaxel arm (S2 Fig).

## Discussion

In oncology drug development, OS is the most relevant endpoint for immunotherapy-based modalities, with many studies definitively demonstrating the disconnect between RECIST response and OS. Given the large number of combination clinical studies currently ongoing in the field of cancer immunotherapy, it becomes imperative to identify early signals of OS to distinguish clinically meaningful benefit in early drug development and accordingly prioritize combinations for expanded development opportunities. This necessitates an understanding of the association of monotherapy CPI with early surrogates of OS.

CRP is an acute phase-reactant protein. Many studies and meta-analyses have shown that elevated preoperative serum CRP is a significant prognostic factor for poor survival compared to patients with normal CRP levels in NSCLC [9, 14]. Recent reports suggest that even in patients with advanced disease, elevated CRP levels correlate with tumor size and staging in NSCLC [15]. We examined CRP in two randomized NSCLC trials, POPLAR and OAK, evaluating monotherapy atezolizumab vs docetaxel with the intent of assessing the association of systemic inflammation with CPI therapy. Longitudinal CRP measurements were performed at a central laboratory. Our data supports the association of elevated serum CRP at baseline with poor clinical outcome independent of treatment. More importantly, we show that CRP modulation post-atezolizumab treatment is associated with response to treatment. The findings from the Phase II POPLAR trial were independently validated in the Phase III trial OAK. CRP decrease 6 weeks post-treatment initiation was associated with PFS and OS benefit in patients treated with atezolizumab in second-line NSCLC, even in patients whose tumors appeared unchanged by RECIST 1.1 (SD patients) at six weeks. Moreover, this association was independent of baseline CRP levels and was associated with CPI and not with chemotherapy.

While associations of CRP with outcome have been reported in similar patient populations in the past, the results have been inconclusive. Chemotherapy related CRP changes may be very specific to the type of chemotherapy and concomitant steroid treatment. In a study of advanced NSCLC patients treated with chemotherapy, n = 142 patients considered evaluable for cancer related CRP were suggested to have better prognosis if CRP levels decreased after two cycles of treatment [16], contrary to our findings for the docetaxel arm (Table 1). One confounding factor may be the steroid pretreatment administered with docetaxel that is known to affect CRP levels [17]. In contrast, in a small study with n = 31 NSCLC patients treated with pembrolizumab monotherapy, the authors found that serum CRP levels at pre-treatment were not predictive, but increase of serum CRP at 6 weeks after anti-PD-1 therapy was predictive of clinical benefit [18]. They also reported that the elevated CRP group showed longer PFS and OS than the depressed CRP group. These results contradict other published reports, and their discrepancy with our observations in POPLAR and OAK may be reflective of the small number of patients in their study. Our two large randomized clinical studies clearly show that decreasing CRP as opposed to an increase in CRP post-atezolizumab treatment is a marker of good outcome for patients on anti-PD-L1 therapy in second line NSCLC and also re-affirms that pre-treatment elevated CRP is a marker of poor prognosis in NSCLC. In both POPLAR and OAK studies, PFS benefit was restricted to the baseline tumor PD-L1 high population as defined by 10% of immune cells or by 50% tumor cells staining positive for PD-L1 [10, 11]. This population represents ~ 16% of NSCLC patients. Using the longitudinal change in CRP as an on-treatment surrogate for clinical benefit, ~ 28% of patients in OAK represent the patient population experiencing PFS benefit. These patients also showed remarkable OS benefit. The patient pool that showed no increase in CRP at 6 weeks also showed OS improvement, further highlighting the value of serum CRP as a surrogate to interpret OS signals in early clinical trials with CPIs. Patients with an increase in serum CRP showed no improvement in OS irrespective of their RECIST based response assessment at six weeks.

CRP belongs to the pentraxin family of calcium dependent ligand-binding plasma proteins and is secreted in the periphery primarily by the liver [19]. There is growing evidence that CRP plays important roles in inflammatory processes and host responses to infection including the complement pathway, apoptosis, phagocytosis, nitric oxide (NO) release, and the production of cytokines, particularly interleukin-6 and tumor necrosis factor-α. Indeed, a remarkable association is observed between serum CRP and plasma IL-6 in patients with NSCLC [20]. CRP induces the upregulation of p53 in monocytes and affects cell cycle kinetics of monocytes through binding to CD32 (FcγRII), inducing apoptosis by G2/M arrest in the cell cycle [21]. FcγRII receptors have been shown to trigger apoptotic signals and are expressed in monocytes that polarize to pro-inflammatory macrophages, suggesting that high CRP may dampen macrophage-driven pro-inflammatory responses by inducing apoptosis [19, 22]. Thus, in conditions of high systemic CRP, there may be a greater likelihood of tumor cells undergoing an ‘immunologically silent’ death with minimal antigen presentation to facilitate priming and activation of effector T cells and may contribute to the lack of efficacy of a PD-L1 blockade.

While the training and test set based evaluation of serial CRP measurement on patient outcomes adds robustness and confidence to our findings, there are a few limitations worth noting. This analysis only included patients that were on study for at least 2 cycles of therapy and could provide serum samples for measurement of CRP and image scans for RECIST measurement. Thus, patients who may have had an early event resulting in no subsequent image scans or serum were excluded from the analysis. Approximately 20% of the ITT population was excluded from analysis for above mentioned reasons. We did conduct a sensitivity analysis to OS using C2D1 (3 weeks post-initiation of study) to address some of these issues and did not observe a strong association between decrease in CRP and improvement in OS. The association with CRP decreases and atezolizumab OS benefit appeared most pronounced at 6 weeks post-treatment initiation and then reduced levels of CRP were maintained in patients showing OS benefit. This may reflect the time taken for systemic changes to manifest post-treatment. Nevertheless, it is possible that there may be an inherent bias that cannot be readily addressed in this study. Lastly, while this association has been made for monotherapy CPI, the association between serial CRP decrease and outcomes to chemotherapy combination regimens with CPI is worth assessing as these are the new standard of care regimens for front-line NSCLC patients [23, 24].

## Conclusions

In conclusion, our data suggest that a 1.5 fold decrease in serum CRP as early as 6 weeks post-atezolizumab monotherapy has the potential to be an early surrogate that can predict for survival benefit to CPI, independent of RECIST1.1 response. This simple assay, used in conjunction with radiographic surveillance, can aid in making informed decisions for the clinical development in cancer immunotherapy.

## Supporting information

S1 Fig CRP decrease is associated with OS post-atezolizumab treatment, even in patients with high CRP levels at baseline. (DOCX)

S2 Fig Receiver operating characteristic (ROC) plots show the ability of CRP decrease (FC<1.5) to predict 1-year OS. (DOCX)

S1 Table Patient demographics in the two treatment arms in OAK. (DOCX)

S2 Table Patient clinical characteristics in the two treatment arms in OAK. (DOCX)

S1 List (PDF)

S2 List Names and addresses of institutional review boards/ethics committees. (PDF)
