# Supplementary material for: C-reactive protein reduction post treatment is associated with improved survival in atezolizumab (anti-PD-L1) treated non-small cell lung cancer patients
Source: PLoS One. 2021 Feb 3;16(2):e0246486. doi: 10.1371/journal.pone.0246486 (PMC7857603; doi:10.1371/journal.pone.0246486)
Supplement: S1 Fig — (DOCX) [file pone.0246486.s001.docx]

**S1 Fig.** **CRP decrease is associated with OS post-atezolizumab treatment, even in patients with high CRP levels at baseline.**


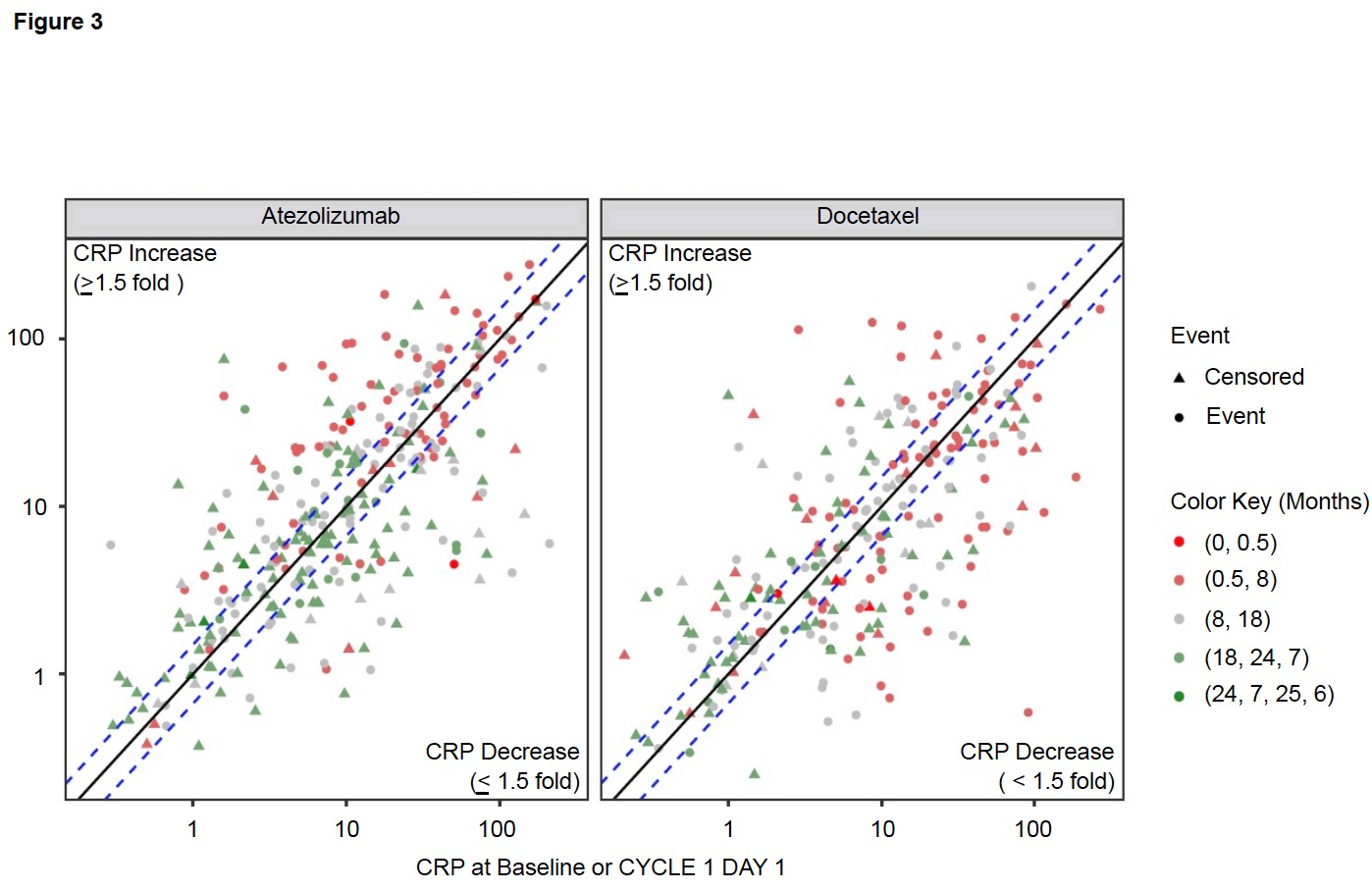


Observed values of CRP from cycle 3 day 1 (C3D1, y axis) vs baseline (x axis) in log scale. Solid black lines indicate no change from baseline. Dashed blue lines indicate increase (1.5 fold increase) or decrease (1.5 fold decrease) from baseline. Each patient is color coded based on their OS (days). Red symbols represent survival benefit (>576 days or 18 months) and teal symbols represent poor survival (<323 days or 10 months). OS time is re-baselined to CRP sample collection day at C3D1 before analysis. OS event time is indicated as solid circles; censored time as solid triangles. CRP, C-reactive protein; OS, overall survival.
