# Supplementary material for: C-reactive protein reduction post treatment is associated with improved survival in atezolizumab (anti-PD-L1) treated non-small cell lung cancer patients
Source: PLoS One. 2021 Feb 3;16(2):e0246486. doi: 10.1371/journal.pone.0246486 (PMC7857603; doi:10.1371/journal.pone.0246486)
Supplement: S2 Fig — (DOCX) [file pone.0246486.s002.docx]

**S2 Fig. Receiver operating characteristic (ROC) plots show the ability of CRP decrease (FC<1.5) to predict 1-year OS.**


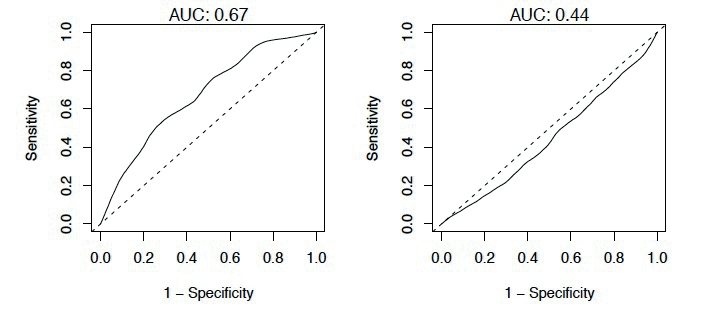


X axis: Probability of CRP ratio larger than a certain cutoff value c among patients who are still alive at 1 year. Y axis: Probability of CRP ratio larger than a certain cutoff value among patients who died before 1 year.
