# Supplementary material for: C-reactive protein reduction post treatment is associated with improved survival in atezolizumab (anti-PD-L1) treated non-small cell lung cancer patients
Source: PLoS One. 2021 Feb 3;16(2):e0246486. doi: 10.1371/journal.pone.0246486 (PMC7857603; doi:10.1371/journal.pone.0246486)
Supplement: S1 Table — (DOCX) [file pone.0246486.s003.docx]

**S1 Table. Patient demographics in the two treatment arms in OAK.**

|  | **All docetaxel** | **BEP Patients with CRP at Baseline and 6 weeks: docetaxel** | **All atezolizumab** | **BEP Patients with CRP  at Baseline and 6 weeks: atezolizumab** |
| --- | --- | --- | --- | --- |
| **AGE** |  |  |  |  |
| N | 425 | 256 | 425 | 302 |
| Mean | 63.3 | 62.92 | 63.06 | 62.92 |
| Median | 64 | 64 | 63 | 63 |
| Min-Max | 34…85 | 36…85 | 33…82 | 35…82 |
| **Bl SLD** |  |  |  |  |
| N | 425 | 256 | 424 | 301 |
| Mean | 76.77 | 72.44 | 76.6 | 73.77 |
| Median | 65 | 62 | 67 | 65 |
| Min-Max | 10…314 | 10…207 | 10…316 | 10…316 |
| NAs | 0 | 0 | 1 | 1 |
| **Race** |  |  |  |  |
| Total | 425 | 256 | 425 | 302 |
| ASIAN | 95 (22.35%) | 62 (24.22%) | 85 (20%) | 59 (19.54%) |
| OTHER | 34 (8%) | 24 (9.38%) | 38 (8.94%) | 28 (9.27%) |
| WHITE | 296 (69.65%) | 170 (66.41%) | 302 (71.06%) | 215 (71.19%) |
| **AGE GROUP** |  |  |  |  |
| Total | 425 | 256 | 425 | 302 |
| >65 | 189 (44.47%) | 109 (42.58%) | 179 (42.12%) | 123 (40.73%) |
| 18 to 65 | 236 (55.53%) | 147 (57.42%) | 246 (57.88%) | 179 (59.27%) |
| **SEX** |  |  |  |  |
| Total | 425 | 256 | 425 | 302 |
| F | 166 (39.06%) | 101 (39.45%) | 164 (38.59%) | 124 (41.06%) |
| M | 259 (60.94%) | 155 (60.55%) | 261 (61.41%) | 178 (58.94%) |
| **HISTOLOGY** |  |  |  |  |
| Total | 425 | 256 | 425 | 302 |
| NON-SQUAMOUS | 315 (74.12%) | 192 (75%) | 313 (73.65%) | 228 (75.5%) |
| SQUAMOUS | 110 (25.88%) | 64 (25%) | 112 (26.35%) | 74 (24.5%) |
| ECOGGR |  |  |  |  |
| Total | 425 | 256 | 425 | 302 |
| 0 | 160 (37.65%) | 109 (42.58%) | 155 (36.47%) | 126 (41.72%) |
| 1 | 265 (62.35%) | 147 (57.42%) | 270 (63.53%) | 176 (58.28%) |

BEP: Biomarker evaluable population; CRP: C-Reactive Protein; BlSLD: Baseline Tumor size or Sum of Longest diameter; ECOG GR: ECOG status; TXC: Treatment Lines; IC Level: PD-L1 on Immune cells (0:<1%; 1:1-5%; 2:>=5-10%; 2: >=10%); TC level: PD-L1 on Tumor cells (0:<1%; 1:1-5%; 2:>=5-50%; 2: >=50%); Mets: Metastases
