# Supplementary material for: C-reactive protein reduction post treatment is associated with improved survival in atezolizumab (anti-PD-L1) treated non-small cell lung cancer patients
Source: PLoS One. 2021 Feb 3;16(2):e0246486. doi: 10.1371/journal.pone.0246486 (PMC7857603; doi:10.1371/journal.pone.0246486)
Supplement: S2 Table — (DOCX) [file pone.0246486.s004.docx]

**S2 Table. Patient clinical characteristics in the two treatment arms in OAK.**

|  | **All docetaxel** | **BEP Patients with CRP at Baseline and 6 weeks: docetaxel** | **All atezolizumab** | **BEP Patients with CRP  at Baseline and 6 weeks: atezolizumab** |
| --- | --- | --- | --- | --- |
| **PRIOR TXC** |  |  |  |  |
| Total | 425 | 256 | 425 | 302 |
| 1 | 320 (75.29%) | 186 (72.66%) | 320 (75.29%) | 225 (74.5%) |
| 2 | 105 (24.71%) | 70 (27.34%) | 105 (24.71%) | 77 (25.5%) |
| **IC LEVEL** |  |  |  |  |
| Total | 421 | 254 | 421 | 300 |
| NAs | 4 | 2 | 4 | 2 |
| 0 | 219 (52.02%) | 129 (50.79%) | 210 (49.88%) | 145 (48.33%) |
| 1 | 142 (33.73%) | 88 (34.65%) | 158 (37.53%) | 117 (39%) |
| 2 | 44 (10.45%) | 26 (10.24%) | 35 (8.31%) | 23 (7.67%) |
| 3 | 16 (3.8%) | 11 (4.33%) | 18 (4.28%) | 15 (5%) |
| **TC LEVEL** |  |  |  |  |
| Total | 421 | 254 | 421 | 300 |
| NAs | 4 | 2 | 4 | 2 |
| 0 | 296 (70.31%) | 174 (68.5%) | 294 (69.83%) | 212 (70.67%) |
| 1 | 18 (4.28%) | 13 (5.12%) | 22 (5.23%) | 17 (5.67%) |
| 2 | 68 (16.15%) | 41 (16.14%) | 65 (15.44%) | 41 (13.67%) |
| 3 | 39 (9.26%) | 26 (10.24%) | 40 (9.5%) | 30 (10%) |
| **SMOKING STATUS** |  |  |  |  |
| Total | 425 | 256 | 425 | 302 |
| CURRENT | 67 (15.76%) | 39 (15.23%) | 59 (13.88%) | 45 (14.9%) |
| NEVER | 72 (16.94%) | 45 (17.58%) | 84 (19.76%) | 59 (19.54%) |
| PREVIOUS | 286 (67.29%) | 172 (67.19%) | 282 (66.35%) | 198 (65.56%) |
| **LIVER METS** |  |  |  |  |
| Total | 425 | 256 | 425 | 302 |
| N | 331 (77.88%) | 206 (80.47%) | 342 (80.47%) | 261 (86.42%) |
| Y | 94 (22.12%) | 50 (19.53%) | 83 (19.53%) | 41 (13.58%) |

BEP: Biomarker evaluable population; CRP: C-Reactive Protein; BlSLD: Baseline Tumor size or Sum of Longest diameter; ECOG GR: ECOG status; TXC: Treatment Lines; IC Level: PD-L1 on Immune cells (0:<1%; 1:1-5%; 2:>=5-10%; 2: >=10%); TC level: PD-L1 on Tumor cells (0:<1%; 1:1-5%; 2:>=5-50%; 2: >=50%); Mets: Metastases NA: Not Assessed
