# Supplementary material for: C-reactive protein reduction post treatment is associated with improved survival in atezolizumab (anti-PD-L1) treated non-small cell lung cancer patients
Source: PLoS One. 2021 Feb 3;16(2):e0246486. doi: 10.1371/journal.pone.0246486 (PMC7857603; doi:10.1371/journal.pone.0246486)
Supplement: S1 List — (PDF) [file pone.0246486.s005.pdf]

Site	Investigator	Address	Ethics Committee / Institutional Review Board Address
258413	Russell DeVore III, M.D.	Center for Biomedical Research LLC, 1415 Old Weisgarber Rd, #200, Knoxville, TN, 37909, UNITED STATES	QUORUM REVIEW IRB, 1501 5th Avenue, Suite 1000, Seattle, WA, 98101, UNITED STATES
258415	Jennifer Suga	Kaiser Permanente - Vallejo, 975 Serono Drive, Vallejo, CA, 94589, UNITED STATES	Kaiser Permanente Northern California IRB, 1800 Harrison Street, 16th Floor, Oakland, CA, 94612, UNITED STATES
258416	Misako Nagasaka	Karmanos Cancer Institute, 87 East Canfield, Detroit, MI, 48201, UNITED STATES	Wayne State University Human Investigation committee, 87 East Canfield, Second Floor, Detroit, MI, 48201, UNITED STATES
258418	Pamela Smith	Billings Clinic; Research Center, 1045 North 30th Street, Billings, MT, 59101, UNITED STATES	QUORUM REVIEW IRB, 1501 5th Avenue, Suite 1000, Seattle, WA, 98101, UNITED STATES
258429	Mark Kozloff, M.D.	Ingalls Memorial Hospital, One Ingalls Drive, W740, Harvey, IL, 60426, UNITED STATES	Ingalls Memorial Hospital Institutional Review Board, C/O Greg Biedron, Pharm. D., Chairman, One Ingalls Drive, Harvey, IL, 60426, UNITED STATES
258438	Jonathan Polikoff M.D.	Kaiser Permanente - San Marcos, 400 Craven Rd, San Marcos, CA, 92069, UNITED STATES	Kaiser Permanente Southern California, 393 East Walnut Street, 2nd Floor, Pasadena, CA, 91188, UNITED STATES
258440	Rodolfo Bordoni M.D.	Georgia Cancer Specialists, 1835 Savoy Drive, Suite 104, Atlanta, GA, 30341, UNITED STATES	QUORUM REVIEW IRB, 1501 5th Avenue, Suite 1000, Seattle, WA, 98101, UNITED STATES
258443	Gary MacVicar	Illinois Cancer Care, 8940 North Wood Sage Road, Peoria, IL, 61615, UNITED STATES	QUORUM REVIEW IRB, 1501 Fourth Ave., Suite 800, Seattle, WA, 98101, UNITED STATES
258527	James R Cunningham	Providence St. Mary Regional Cancer Center, 401 West Poplar Street, Walla Walla, WA, 99362, UNITED STATES	QUORUM REVIEW IRB, 1501 5th Avenue, Suite 1000, Seattle, WA, 98101, UNITED STATES
258535	Humera Khurshid	Rhode Island Hospital, 593 Eddy Street, Providence, RI, 02903, UNITED STATES	Rhode Island Hospital IRB, One Hoppin Street, Coro Building West Suite 1.300, Providence, RI, 02903, UNITED STATES
258540	Thomas Christian M.D.	New England Cancer Specialists, 100 Campus Drive, Unit 108, Scarborough, ME, 04074, UNITED STATES	QUORUM REVIEW IRB, 1501 5th Avenue, Suite 1000, Seattle, WA, 98101, UNITED STATES
258690	Aleksandra Szczesna M.D.	Mazowieckie Centrum Leczenia Chorob Pluc I Gruzlicy; Oddzial III, III Oddzial Chorob Pluc z,	Komisja Bioetyczna przy Centrum Onkologii - Intytucie im. Marii Skłodowskiej-Curie, UL. Roentgena 5, 02-781,

Site	Investigator	Address	Ethics Committee / Institutional Review Board Address
	PhD	pododdzialem Onkologicznym; ul. Reymonta 83/91, 05-400, Otwock, POLAND	Warsaw, POLAND
258691	Dr. Dariusz Kowalski	Centrum Onkologii – Instytut im. Marii Skłodowskiej-Curie Klinika Nowotworów Piersi i Chirurgii Reko, UL. Roentgena 5, 02-781, Warszawa, POLAND	Komisja Bioetyczna przy Centrum Onkologii - Intytucie im. Marii Skłodowskiej-Curie, UL. Roentgena 5, 02-781, Warsaw, POLAND
258692	Rafal Dziadziuszko	Uniwersyteckie Centrum Kliniczne, Klinika Onkologii i Radioterapii, Ul. Debinki 7, 80-952, Gdansk, POLAND	Komisja Bioetyczna przy Centrum Onkologii - Intytucie im. Marii Skłodowskiej-Curie, UL. Roentgena 5, 02-781, Warsaw, POLAND
258693	Igor Symonowicz	Wojewodzki Szpital Specjalistyczny im. M. Kopernika;Oddzial Chemioterapii, Ul. Pabianicka 62, 93-513, Lodz, POLAND	Komisja Bioetyczna przy Centrum Onkologii - Intytucie im. Marii Skłodowskiej-Curie, UL. Roentgena 5, 02-781, Warsaw, POLAND
258808	Luca Gianni M.D.	Irccs Ospedale San Raffaele;Oncologia Medica, Via Olgettina 60, 20132, Milano, Lombardia, ITALY	Comitato Etico Irccs Ospedale San Raffaele, Via Olgettina 60, 20132, Milano, Lombardia, ITALY
258829	Assoc. Prof. Hasan Senol Coskun	Akdeniz University Medical Faculty; Medical Oncology Department, Akdeniz Üniversitesi Tıp Fakültesi Tıbbi Onkoloji Bilim Dalı H Blok 1. Kat Antalya, 07070, Antalya, TURKEY	Akdeniz University, Dumlupinar Bulvari, 07059, Antalya, TURKEY
259037	Eli Kirshner M.D.	The Valley Hospital, 1124 E. Ridgewood Avenue, Office of Clinical Trials, Ridgewood, NJ, 07450, UNITED STATES	Western IRB, 3535 Seventh Ave, SW, Olympia, WA, 98502, UNITED STATES
259075	Dr Cesare Gridelli	Citta Ospedaliera; Divisione Oncologia Medica, Contrada Amoretta, 83100, Avellino, Campania, ITALY	Comitato Etico Dell'Az. Osp. S Giuseppe Moscati, Contrada Amoretta Citta Ospedaliera, 83100, Avellino, Campania, ITALY
259178	Dr. Johan Vansteenkiste	UZ Leuven, Campus Gasthuisberg, Herestraat 49, 3000, Leuven, BELGIUM	Commissie Medische Ethiek UZ Leuven, Campus Gasthuisberg E330, Herestraat 49, 3000, Leuven, BELGIUM
259224	Danielle Power	Charing Cross Hospital; Medical Oncology, Fulham Palace Road, London, W6 8RF, UNITED KINGDOM	NRES Committee London – Surrey Borders, 80 London Road Ground floor, Skipton House, London, SE1 6LH, UNITED KINGDOM
259225	Dr Yvonne Summers	Christie Hospital NHS Trust; Medical Oncology, Wilmslow Road, Withington, Lancashire, Manchester, M2O 4BX, UNITED	The Christie NHS Foundation Trust; Research & Development Office, Block C, Withington Hall,Wilmslow Road, Withington, Manchester, M20 4BX, UNITED

Site	Investigator	Address	Ethics Committee / Institutional Review Board Address
		KINGDOM	KINGDOM
259226	Dr James Spicer	Guys and St Thomas NHS Foundation Trust, Guys Hospital, Great Maze Pond, London, SE1 9RT, UNITED KINGDOM	Joint Clinical Trials Office; R&D., 16th Floor Tower Wing, Guy's Hospital, Great Maze Pond, London, SE1 9RT, UNITED KINGDOM
259231	Ekaterini Boleti	Royal Free Hospital; Dept of Oncology, Pond Street, London, NW3 2QG, UNITED KINGDOM	NRES Committee London – Surrey Borders, 80 London Road Ground floor, Skipton House, London, SE1 6LH, UNITED KINGDOM
259300	Paul R Conkling M.D.	Virginia Oncology Associates, 5900 Lake Wright Drive, Norfolk, VA, 23502, UNITED STATES	US Oncology, Inc Institutional Review Board, 10101 Woodloch Forest, The Woodlands, TX, 77380, UNITED STATES
259301	Joseph Fiorillo M.D.	Willamette Valley Cancer Ctr - 520 Country Club, 520 Country Club Road, Eugene, OR, 97401- 8122, UNITED STATES	US Oncology, Inc Institutional Review Board, 10101 Woodloch Forest, The Woodlands, TX, 77380, UNITED STATES
259305	Robert Jotte M.D.	Rocky Mountain Cancer Centers - Colorado Springs (Circle), 3027 North Circle Dr., Colorado Springs, CO, 80909, UNITED STATES	US Oncology, Inc Institutional Review Board, 10101 Woodloch Forest, The Woodlands, TX, 77380, UNITED STATES
259306	Rama Balaraman	Ocala Oncology Center, 433 Sw 10th Street, Ocala, FL, 34471, UNITED STATES	US Oncology, Inc Institutional Review Board, 10101 Woodloch Forest, The Woodlands, TX, 77380, UNITED STATES
259308	Stephen Richey	Texas Oncology, P.A. - Fort Worth, 1001 12th Ave, #200, Fort Worth, TX, 76104, UNITED STATES	US Oncology, Inc Institutional Review Board, 10101 Woodloch Forest, The Woodlands, TX, 77380, UNITED STATES
259309	Richard Rosenberg	Arizona Oncology Associates, PC - Hope, 1521 East Tangerine Rd, Building 3, Suite 130, Oro Valley, AZ, 85755, UNITED STATES	US Oncology, Inc Institutional Review Board, 10101 Woodloch Forest, The Woodlands, TX, 77380, UNITED STATES
259311	Fadi Braiteh	Comprehensive Cancer Centers of Nevada – Eastern Avnue, 3730 S. Eastern Avenue, Las Vegas, NV, 89169, UNITED STATES	US Oncology, Inc Institutional Review Board, 10101 Woodloch Forest, The Woodlands, TX, 77380, UNITED STATES
259312	David A Smith M.D.	Northwest Cancer Specialists - Vancouver, 210 SE 136th Avenue, Vancouver, WA, 98684, CANADA	US Oncology, Inc Institutional Review Board, 10101 Woodloch Forest, The Woodlands, TX, 77380, UNITED STATES

Site	Investigator	Address	Ethics Committee / Institutional Review Board Address
259313	Alexander I Spira M.D.	Virginia Cancer Specialists, PC, 8503 Arlington Boulevard, Suite 400, Fairfax, VA, 22031, UNITED STATES	US Oncology, Inc Institutional Review Board, 10101 Woodloch Forest, The Woodlands, TX, 77380, UNITED STATES
259314	James Uyeki M.D.	Texas Oncology - South Austin, 4101 James Casey, Suite 100, Austin, TX, 78745, UNITED STATES	US Oncology, Inc Institutional Review Board, 10101 Woodloch Forest, The Woodlands, TX, 77380, UNITED STATES
259315	Lawrence Garbo M.D.	New York Oncology Hematology, P.C., 400 Patroon Creek Blvd, Suite One, Albany, NY, 12206, UNITED STATES	US Oncology, Inc Institutional Review Board, 10101 Woodloch Forest, The Woodlands, TX, 77380, UNITED STATES
259344	Dr. Victor Cohen	McGill University; Sir Mortimer B Davis Jewish General Hospital; Oncology, 3755 Côte-Ste Catherine, E1031, H3T 1E2, Montreal, Quebec, CANADA	McGill University; McGill University; Ethics Board, 3655 Promenade Sir William Osler - 6th Floor, H3G 1Y6, Montreal, Quebec, CANADA
259352	Hande Turna	Istanbul Uni Cerrahpasa Medical Faculty Hospital; Medical Oncology, Istanbul Universitesi Cerrahpasa Tip Fakultesi, Medikal Onkoloji Bilim Dali, Cerrahpasa, 34300, Istanbul, TURKEY	Akdeniz University, Dumlupinar Bulvari, 07059, Antalya, TURKEY
259396	Dr. Charuwan Akewanlop	Faculty of Med. Siriraj Hosp.; Med.-Div. of Med. Oncology, 2 Prannok Road, 13 Fl. Chalermprakiat Building, 10700, Bangkok, THAILAND	Siriraj Institutional Review Board, Mahidol University, Siriraj H., His Majesty the King's 80th Birthday, Buidling, Room 210, 2 Prannok road, Siriraj, 10700, Bangkoknoi, THAILAND
259397	Associate Professor Virote Sriuranpong	Chulalongkorn Hospital; Medical Oncology, Rama IV Road, Patumwan, 10400, Bangkok, THAILAND	Institutional Review Board, Faculty of Medicine, 3rd floor, Ananda Mahidol Building,, 10330, Patumwan, Bangkok, THAILAND
259400	Sudsawat Laohavinij	Rajavithi Hospital; Division of Medical Oncology, 2 Phayathai Road, Rajavithi, 10400, Bangkok, THAILAND	IHRP Ethics Committee; Dept. of Medical Science, Building 8, Floor 7, Room 702-703, Tiwanon Rd., Amphur Muang, Ministry Public Health, 11000, Nonthaburi, THAILAND
259404	Prof Keunchil Park	Samsung Medical Centre; Division of Hematology/Oncology, 81, Irwon-ro, Gangnamgu, 135-710, Seoul, REPUBLIC OF KOREA	Samsung Medical Center; IRB, 81, Irwon-ro, Gangnam-Gu, IRB office, M3, Main building, 135-710, Seoul, REPUBLIC OF KOREA

Site	Investigator	Address	Ethics Committee / Institutional Review Board Address
259406	Byoung Chul Cho	Severance Hospital, Yonsei University Health System, 50 Yonsei-ro, Seodaemun-gu, 120-752, Seoul, REPUBLIC OF KOREA	SeveranceHospital- YonseiUniversity; IRB, 50, Yonsei-ro, Seodaemun-gu, 120-752, Seoul, REPUBLIC OF KOREA
259408	Ji-Youn Han	National Cancer Center; Medical Oncology, 323 Ilsan-ro, Ilsandong-gu, 410-769, Gyeonggi-do, REPUBLIC OF KOREA	National Cancer Center Institutional Review Board, 323 Ilsanro, Ilsandong-gu, 410-769, Goyang-si, REPUBLIC OF KOREA
259427	Ronald Harris	Broome Oncology - Binghamton, 169 Riverside Drive, Suite 209, Binghamton, NY, 13905, UNITED STATES	US Oncology, Inc Institutional Review Board, 10101 Woodloch Forest, The Woodlands, TX, 77380, UNITED STATES
259431	Jerome Goldschmidt-Jr M.D.	Blue Ridge Cancer Care, 2600 Research Center Drive, Suite 5, Blacksburg, VA, 24060, UNITED STATES	US Oncology, Inc Institutional Review Board, 10101 Woodloch Forest, The Woodlands, TX, 77380, UNITED STATES
259571	Anders Vikström	Universitetssjukhuset Linköping; Lungmedicinkliniken, Universitetssjukhuset Linköping, 581 85, Linköping, SWEDEN	Regionala etikprövningsnämnden i Linköping, c/o Hälsouniversitetets kansli, Sandbäcksgatan 7, SE-581 83, Linköping, SWEDEN
260081	Reginald Comeau	Cite De La Sante De Laval; Hemato-Oncologie, 1755 Boul. Rene-Laennec, H7M 3L9, Laval, Quebec, CANADA	Cite De La Sante De Laval, 1755 Rene-Laennec, H7M 3L9, Laval, Quebec, CANADA
260319	Joachim von Pawel, M.D.	Asklepios-Fachkliniken Muenchen-Gauting; Onkologie, Robert-Koch-Allee 2, 82131, Gauting, GERMANY	Ethik-Kommission des Landes Sachsen-Anhalt, Kühnauer Str. 70, 06846, Dessau-Roßlau, GERMANY
260320	Dr. Achim Rittmeyer	Fachklinik für Lungenerkrankungen, Robert-Koch-Str. 3, 34376, Immenhausen, GERMANY	Ethik-Kommission des Landes Sachsen-Anhalt, Kühnauer Str. 70, 06846, Dessau-Roßlau, GERMANY
260321	Dr. med. Wolfgang Schütte, CA PD	Krankenhaus Martha-Maria Halle-Doelau gGmbH; Klinik fuer Innere Medizin II, Roentgenstr. 1, 06120, Halle, GERMANY	Ethik-Kommission des Landes Sachsen-Anhalt, Kühnauer Str. 70, 06846, Dessau-Roßlau, GERMANY
260322	Prof. Dr. Christian Schulz	Universitätsklinikum Regensburg; Klinik und Poliklinik für Innere Medizin II, Pneumologie, Franz-Josef-Strauß-Allee 11, 93053, Regensburg, GERMANY	Ethik-Kommission des Landes Sachsen-Anhalt, Kühnauer Str. 70, 06846, Dessau-Roßlau, GERMANY
260875	Santiago Ponce Aix	Hospital Universitario 12 de Octubre; Servicio de Oncologia, Avenida Cordoba Km 5.4, Edificio Materno- Infantil 2ª planta, 28041, Madrid, SPAIN	Hospital Ramon y Cajal ;Comité Etico de Investigación Clínica, Ctra. Colmenar Viejo, km 9,1, 28034, Madrid, SPAIN

Site	Investigator	Address	Ethics Committee / Institutional Review Board Address
260876	Dr. Javier R De Castro Carpeno	Hospital La Paz, Paseo de la Castellana 261, Planta baja, Edificio escuela de Enfermería, 28046, Madrid, SPAIN	Hospital Ramon y Cajal ;Comité Etico de Investigación Clínica, Ctra. Colmenar Viejo, km 9,1, 28034, Madrid, SPAIN
260877	Pilar Garrido Lopez, M.D.	Hospital Ramon y Cajal; Servicio de Oncologia, Ctra. Colmenar Viejo, km 9,1, planta -2 derecha, 28034, Madrid, SPAIN	Hospital Ramon y Cajal ;Comité Etico de Investigación Clínica, Ctra. Colmenar Viejo, km 9,1, 28034, Madrid, SPAIN
260878	Angel Artal Cortes, M.D.	Hospital Universitario Miguel Servet; Servicio Oncologia, Paseo Isabel La Catolica 1-3, 50009, Zaragoza, SPAIN	Hospital Ramon y Cajal ;Comité Etico de Investigación Clínica, Ctra. Colmenar Viejo, km 9,1, 28034, Madrid, SPAIN
261288	Dr Henri Janicot	Hopital Gabriel Montpied; Service De Pneumologie, 58 Rue Montalembert, 63003, Clermont-Ferrand, FRANCE	CPP Sud Ouest et Outre Mer I, 10 chemin du raisin, ARS Midi-Pyrénées, Bureau 1028, 31050, Toulouse cedex 9, France
261289	Gwenaelle Le Garff	Centre Hospitalier de Saint Brieuc - Hôpital Yves Le Foll; Pneumologie, 10 Rue Marcel Proust, Cedex 1, 22027, St Brieuc, FRANCE	CPP Sud Ouest et Outre Mer I, 10 chemin du raisin, ARS Midi-Pyrénées, Bureau 1028, 31050, Toulouse cedex 9, France
261290	Dr Julien Mazieres	Hopital Larrey; Pneumologie, 24 Chemin De Pouvourville, Cedex 9, 31059, Toulouse, FRANCE	CPP Sud Ouest et Outre Mer I, 10 chemin du raisin, ARS Midi-Pyrénées, Bureau 1028, 31050, Toulouse cedex 9, FRANCE
261291	Dr Denis Moro-Sibilot	Hôpital Nord Michallon; Pneumologie, Avenue des Maquis du Gresivaudan, 38700, La Tronche, FRANCE	CPP Sud Ouest et Outre Mer I, 10 chemin du raisin, ARS Midi-Pyrénées, Bureau 1028, 31050, Toulouse cedex 9, FRANCE
261292	Philippe Scheid	Centre D'oncologie De Gentilly; Oncology, 2 Rue Marie Marvingt, 54100, Nancy, FRANCE	CPP Sud Ouest et Outre Mer I, 10 chemin du raisin, ARS Midi-Pyrénées, Bureau 1028, 31050, Toulouse cedex 9, FRANCE
264493	Raul Mena M.D.	Innovative Clinical Research Institute, 15111 E. Whittier Blvd., Suite 216, Whittier, CA, 90603, UNITED STATES	QUORUM REVIEW IRB, 1501 Fourth Ave., Suite 800, Seattle, WA, 98101, UNITED STATES
