# Supplementary material for: C-reactive protein reduction post treatment is associated with improved survival in atezolizumab (anti-PD-L1) treated non-small cell lung cancer patients
Source: PLoS One. 2021 Feb 3;16(2):e0246486. doi: 10.1371/journal.pone.0246486 (PMC7857603; doi:10.1371/journal.pone.0246486)
Supplement: S2 List — (PDF) [file pone.0246486.s006.pdf]

Names and Addresses of Institutional Review Boards / Ethics Committees
Protocol: GO28915

Site #	Investigator	IRB/IEC Name and Address	Approval Date
260347	Lingua, Alejo	Comite Institucional de Etica de Investigacion en Salud (C.I.E.I.S.), Av. Naciones unidas 346, 5016, Cordoba, ARGENTINA	13-Aug-2014
263540	Syrigos, Konstantinos	National Ethics Committee, Ministry of Health and Social Wellfare, 284, Messogion Avenue, 15562, Cholargos, GREECE	30-Jun-2014
263541	KALOFONOS, HARALABOS	National Ethics Committee, Ministry of Health and Social Wellfare, 284, Messogion Avenue, 15562, Cholargos, GREECE	30-Jun-2014
263564	Helland, Aslaug	REK sør-øst, Posboks 1130. Blindern, 0318, Oslo, NORWAY	19-Mar-2014
263595	CHITNENI, SHOBHA	QUORUM REVIEW IRB, 1501 Fourth Ave., Suite 800, Seattle, WA, 98101, UNITED STATES	11-Dec-2013
263596	Ali, Muhammad-Amjad	QUORUM REVIEW IRB, 1501 Fourth Ave., Suite 800, Seattle, WA, 98101, UNITED STATES	19-Feb-2014
263597	Braiteh, Fadi	US Oncology, Inc Institutional Review Board, 10101 Woodloch Forest, The Woodlands, TX, 77380, UNITED STATES	29-Jan-2014
263598	CONKLING, PAUL R.	US Oncology, Inc Institutional Review Board, 10101 Woodloch Forest, The Woodlands, TX, 77380, UNITED STATES	29-Jan-2014
263602	FEHRENBACHER, LOUIS	Kaiser Permanente Northern California IRB, 1800 Harrison Street, 16th Floor, Oakland, CA, 94612, UNITED STATES	18-Mar-2014
263603	Fiorillo, Joseph	US Oncology, Inc Institutional Review Board, 10101 Woodloch Forest, The Woodlands, TX, 77380, UNITED STATES	29-Jan-2014
263605	Gadgeel, Shirish	Wayne State University Human Investigation committee, 87 East Canfield, Second Floor, Detroit, MI, 48201, UNITED STATES	21-May-2014
263606	Gandara, David	UC Davis IRB, 2921 Stockton Blvd., Suite 1429, Sacramento, CA, 95817, UNITED STATES	10-Apr-2014
263610	Kabbinavar, Fairooz	Office of the Human Research; Protection Program, 11000 Kinross Ave., Ste. 211, PO Box 951694, Los Angeles, CA, 90095, UNITED STATES	11-Mar-2014
263611	Khurshid, Humera	Rhode Island Hospital IRB, One Hoppin Street, Coro Building West Suite 1.300, Providence, RI, 02903, UNITED STATES	14-May-2014
263613	Kirshner, Eli	Western Institutional Review Board WIRB Panel 7, 1019 39th Ave S.E., Puyallup, WA, 98374, UNITED STATES	03-Mar-2014
263614	Kozloff, Mark	Ingalls Memorial Hospital Institutional Review Board, C/O Greg Biedron, Pharm. D., Chairman, One Ingalls Drive, Harvey, IL, 60426, UNITED STATES	06-Dec-2013
263618	Mullane, Michael	Aurora HealthCare; Institutional Review Board, 945 N 12th St, Milwaukee, WI, 53233, UNITED STATES	14-Mar-2014
263620	Smith, Pamela	QUORUM REVIEW IRB, 1501 Fourth Ave., Suite 800, Seattle, WA, 98101, UNITED STATES	02-Dec-2013
263624	POLIKOFF, JONATHAN	Kaiser Permanente Southern California, 393 East Walnut Street, 2nd Floor, Pasadena, CA, 91188, UNITED STATES	21-Jan-2014
263625	Rose, Andrea	QUORUM REVIEW IRB, 1501 Fourth Ave., Suite 800, Seattle, WA, 98101, UNITED STATES	09-Dec-2013
263627	Bordoni, Rodolfo	QUORUM REVIEW IRB, 1501 Fourth Ave., Suite 800, Seattle, WA, 98101, UNITED STATES	24-Mar-2014
263629	Smith, David A.	US Oncology, Inc Institutional Review Board, 10101 Woodloch Forest, The Woodlands, TX, 77380, UNITED STATES	29-Jan-2014

Site #	Investigator	IRB/IEC Name and Address	Approval Date
263630	Spira, Alexander I.	US Oncology, Inc Institutional Review Board, 10101 Woodloch Forest, The Woodlands, TX, 77380, UNITED STATES	29-Jan-2014
263631	MacVicar, Gary	QUORUM REVIEW IRB, 1501 Fourth Ave., Suite 800, Seattle, WA, 98101, UNITED STATES	27-Dec-2013
263632	THOMAS, CHRISTIAN	QUORUM REVIEW IRB, 1501 Fourth Ave., Suite 800, Seattle, WA, 98101, UNITED STATES	27-Jan-2014
263633	Uyeki, James	US Oncology, Inc Institutional Review Board, 10101 Woodloch Forest, The Woodlands, TX, 77380, UNITED STATES	29-Jan-2014
263638	Patel, Manish	Human Research Protections Program, 9500 Gilman Drive, Mail Code 0052, La Jolla, CA, 92093-0052, UNITED STATES	24-Jun-2014
263682	Tiseo, Marcello	Comitato Etico per Parma, Via Gramsci, 14, 43126, Parma, Emilia-Romagna, ITALY	16-Jul-2014
263685	Cortinovis, Diego	CE DELLA PROVINCIA MONZA BRIANZA C/O UFFICIO SPERIMENTAZIONI CLINICHE AZ OSPEDALIERA "S. GERARDO", VIA PERGOLESI 33, 20052, MONZA, Lombardia, ITALY	08-May-2014
263687	DE MARINIS, FILIPPO	Comitato Etico Degli IRCCS Istituto Europeo di Oncologia e Centro Cardiologico Monzino, VIA RIPAMONTI 435, 20141, MILANO, Lombardia, ITALY	07-May-2014
263688	Follador, Alessandro	Comitato Etico Regionale Unico CERU, P.le S. Maria della Misericordia, 15, Pad 10 - 3° Piano, 33100, Udine, Friuli-Venezia Giulia, ITALY	03-Jun-2014
263689	GIANNI, LUCA	Comitato Etico Irccs Ospedale San Raffaele, VIA OLGETTINA 60, 20132, MILANO, Lombardia, ITALY	06-Mar-2014
263691	GRIDELLI, CESARE	COMITATO ETICO DELL'AZ. OSP. S GIUSEPPE MOSCATI, CONTRADA AMORETTA CITTA OSPEDALIERA, 83100, AVELLINO, Campania, ITALY	22-Oct-2014
263695	Gautschi, Oliver	Ethikkommission Nordwest- und Zentralschweiz (EKNZ), Hebelstrasse 53, 4056, Basel, SWITZERLAND	30-Apr-2014
263699	Mach, Nicolas	Commission centrale d'éthique, Hôpital Universitaire Genève, Rue Gabrielle-Perret-Gentil 4, 1211, Genève, SWITZERLAND	17-Feb-2014
263700	Waibel, Christine	Ethikkommission Nordwest- und Zentralschweiz (EKNZ), Hebelstrasse 53, 4056, Basel, SWITZERLAND	30-Apr-2014
263785	De Azevedo, Sergio	Comite de Etica do Hospital de Clinicas de Porto Alegre, Rua Ramiro Barcelos, 2350, 90035-903, Porto Alegre, RS, BRAZIL	28-Jul-2014
263807	Rodrigues, Ana	CEIC - Comissão de Ética para Investigação Clínica, CEIC - Comissão de Ética para Investigação Clínica, Av. do Brasil, 53 - Pav 17-A ,Parque da Saúde de Lisboa, 1749-004, Lisboa, PORTUGAL	04-Apr 2014
263809	Teixeira, Encarnaçao	CEIC - Comissão de Ética para Investigação Clínica, CEIC - Comissão de Ética para Investigação Clínica, Av. do Brasil, 53 - Pav 17-A ,Parque da Saúde de Lisboa, 1749-004, Lisboa, PORTUGAL	04-Apr 2014
263811	BARATA, FERNANDO	CEIC - Comissão de Ética para Investigação Clínica, CEIC - Comissão de Ética para Investigação Clínica, Av. do Brasil, 53 - Pav 17-A ,Parque da Saúde de Lisboa, 1749-004, Lisboa, PORTUGAL	04-Apr 2014
263884	Kono, Scott	Kaiser Permanente Colorado Institutional Review Board, 10065 East Harvard Avenue, Suite 300, Denver, CO, 80231, UNITED STATES	23-Jan-2014
263893	Chen, Hongbin	Institutional Review Board, ELM & CARLTON STREETS, BUFFALO, NY, 14263, UNITED STATES	10-Jun-2014

Site #	Investigator	IRB/IEC Name and Address	Approval Date
263898	Knapp, Mark	QUORUM REVIEW IRB, 1501 Fourth Ave., Suite 800, Seattle, WA, 98101, UNITED STATES	27-Nov-2013
263905	LEACH, JOSEPH	US Oncology, Inc Institutional Review Board, 10101 Woodloch Forest, The Woodlands, TX, 77380, UNITED STATES	29-Jan-2014
263909	MENA, RAUL	QUORUM REVIEW IRB, 1501 Fourth Ave., Suite 800, Seattle, WA, 98101, UNITED STATES	11-Dec-2013
264010	Rodrigues, Gotardo	QUORUM REVIEW IRB, 1501 Fourth Ave., Suite 800, Seattle, WA, 98101, UNITED STATES	19-Nov-2013
264102	Gupta, Rasna	Ontario Cancer Research Ethics Board, MaRS Centre, South Tower, 101 College Street, Suite 500, M5G 1L7, Toronto, Ontario, CANADA	24-Jun-2014
264132	COHEN, VICTOR	Jewish General Hosptial, 3755 Cote St-Catherine Road, A:925, Montreal, Montreal, Quebec, CANADA	03-Apr-2014
264135	Comeau, Reginald	Comité scientifique et d'éthique de la recherche/CISSL-Hôpital de la Cité-de-la-Santé, 1755 Rene-Laennec, local B-S080, H7M 3L9, Laval, Quebec, CANADA	05-May-2014
264158	Telivala, Bijoy	QUORUM REVIEW IRB, 1501 Fourth Ave., Suite 800, Seattle, WA, 98101, UNITED STATES	11-Dec-2013
264385	KARASEVA, NINA	City Clinical Oncology Dispensary; Onco, 56, Prospect Veteranov, Saint-Petersburg, RUSSIAN FEDERATION	06-May-2014
264387	SMOLIN, ALEXEY	Ethics Committee of the Main Military Clinical Hospital n.a. N.N.Burdenko, 3 Gospitalnaya square, 105229, Moscow, RUSSIAN FEDERATION	23-Apr-2014
264399	BONDARENKO, IGOR	Ethics Committee of Dnipropetrovsk City Multilat. Clinical Hospital №4, Blyzhnya street, 31, 49102, Dnipropetrovsk, UKRAINE	19-Jun-2014
264401	HOTKO, YEVHEN	Ethics Committee of Central City Clinical Hospital, 20 Hryboyedova str., 88000, Uzhgorod, KIEV GOVERNORATE, UKRAINE	12-Jun-2014
264684	BIESMA, B.	Stichting BEBO, Stationsstraat 9, 9401 KV Assen, NETHERLANDS	15-Apr-2014
264696	van den Borne, B.E.E.M.	Stichting BEBO, Stationsstraat 9, 9401 KV Assen, NETHERLANDS	15-Apr-2014
264698	Herder, G.J.M.	Stichting BEBO, Stationsstraat 9, 9401 KV Assen, NETHERLANDS	15-Apr-2014
264865	GREIL, RICHARD	Ethikkommission für das Bundesland Salzburg Sebastian-Stief-Gasse 2, A-5010 Salzburg, AUSTRIA	19-May-2014
264866	HASLBAUER, FERDINAND	Ethikkommission für das Bundesland Salzburg Sebastian-Stief-Gasse 2, A-5010 Salzburg, AUSTRIA	19-May-2014
264867	Pall, Georg	Ethikkommission für das Bundesland Salzburg Sebastian-Stief-Gasse 2, A-5010 Salzburg, AUSTRIA	19-May-2014
264891	Beatty, Patrick	QUORUM REVIEW IRB, 1501 Fourth Ave., Suite 800, Seattle, WA, 98101, UNITED STATES	09-Dec-2013
264893	Goldschmidt-Jr, Jerome	US Oncology, Inc Institutional Review Board, 10101 Woodloch Forest, The Woodlands, TX, 77380, UNITED STATES	04-Mar-2014
264895	Hauke, Ralph	US Oncology, Inc Institutional Review Board, 10101 Woodloch Forest, The Woodlands, TX, 77380, UNITED STATES	29-Jan-2014
264903	RICHARDS, DONALD	US Oncology, Inc Institutional Review Board, 10101 Woodloch Forest, The Woodlands, TX, 77380, UNITED STATES	29-Jan-2014
264907	Weissman, Charles	US Oncology IRB, 10101 Woodloch Forest Drive, The Woodlands, TX, 77380, UNITED STATES	29-Jan-2014

Site #	Investigator	IRB/IEC Name and Address	Approval Date
264912	Arauz, Erick	Comite Nacional de Bioetica de la Investigacion, Ave. Justo Arosemena y Calle 35, 0816-02593, Panama City, PANAMA	21-May-2014
264913	CASTRO-SALGUERO, HUGO	Comité de Ética Independiente Zugueme, 3a Calle 11-36, Zona 15, 01015, Guatemala, GUATEMALA	05-Mar-2014
265018	Bergman, Bengt	Regionala Etikprövningsnämnden i Göteborg, Box 100, Medicinaregatan 3, 405 30, Göteborg, SWEDEN	21-May-2014
265031	SULLIVAN, RICHARD	Northern A HDEC; Ministry of Health, Freyberg Building, 20 Aitken Street, Wellington, 6011, NEW ZEALAND	18-Dec-2013
265032	Srivastava, Archana	Northern A HDEC; Ministry of Health, Freyberg Building, 20 Aitken Street, Wellington, 6011, NEW ZEALAND	18-Dec-2013
265033	MCLAREN, BLAIR	Northern A HDEC; Ministry of Health, Freyberg Building, 20 Aitken Street, Wellington, 6011, NEW ZEALAND	18-Dec-2013
265085	Koivunen, Jussi	PPSHP:n alueellinen eettinen toimikunta, Yhtymähallinto, P.O. Box 10, 90029 Oys, FINLAND	24-Feb-2014
265088	Chu, Quincy	Alberta Cancer Research Ethics Committee (ACREC), 8440-112TH STREET, T6G 2B7, EDMONTON, Alberta, CANADA	07-Apr-2014
265089	Kosty, Michael	Scripps Health IRB, 11025 N. Torrey Pines Rd., Suite 200, La Jolla, CA, 92037, UNITED STATES	07-May-2014
265095	Maasilta, Paula	PPSHP:n alueellinen eettinen toimikunta, Yhtymähallinto, P.O. Box 10, 90029 Oys, FINLAND	24-Feb-2014
265096	Ahvonen, Jarkko	PPSHP:n alueellinen eettinen toimikunta, Yhtymähallinto, P.O. Box 10, 90029 Oys, FINLAND	24-Feb-2014
265522	Bernicker, Eric	The Methodist Hospital Research Institute IRB, 6670 Bertner, Houston, TX, 77030, UNITED STATES	27-Mar-2014
265553	Vinnyk, Yuriy	Ethics Committee of Karkiv Regional Oncology Center, 4 Lesoparkovaja str, 61070, Kharkiv, KHARKIV GOVERNORATE, UKRAINE	18-Jul-2014
265576	PAPISH, STEVEN	QUORUM REVIEW IRB, 1501 Fourth Ave., Suite 800, Seattle, WA, 98101, UNITED STATES	27-Nov-2013
265935	Turna, Hande	Istanbul Uni. Cerrahpasa Tip Fak. Etik Kurulu; Cerrahpasa Tip Fak. Etik Degerlendirme Kurulu, Dekanlik Binasi, Fatih, 34098, Istanbul, TURKEY	06-May-2014
265936	Komurcuoglu, Berna	Istanbul Uni. Cerrahpasa Tip Fak. Etik Kurulu; Cerrahpasa Tip Fak. Etik Degerlendirme Kurulu, Dekanlik Binasi, Fatih, 34098, Istanbul, TURKEY	06-May-2014
265971	PARK, KEUNCHIL	Samsung Medical Center EC, 81, Irwon-ro, Gangnam-gu, 06351, Seoul, KOREA, REPUBLIC OF	21-Jan-2014
265972	Cho, Byoung Chul	SeveranceHospital- YonseiUniversity; IRB, 50, Yonsei-ro, Seodaemun-gu, 03722, Seoul, KOREA, REPUBLIC OF	10-Jan-2014
265973	Han, Ji-Youn	IRB of National Cancer Center, 323, Ilsan-ro, Ilsandong-gu, Goyang-si, 10408, Gyeonggi-do, KOREA, REPUBLIC OF	03-Feb-2014
265974	LEE, JONG-SEOK	Seoul National University Bundang Hospital IRB, 82, Gumi-Ro 173 Beon-Gil, Bundang-Gu, 463-707, Seongnam-Si, Gyeonggi-Do, KOREA, REPUBLIC OF	23-Jan-2014
265975	Kang, Jin-Hyoung	Seoul St. Mary's Hospital; IRB, 222, Banpo-daero, Seocho-gu, 06591, Seoul, KOREA, REPUBLIC OF	27-Jan-2014
265976	Kim, Dong-Wan	Seoul National University Hospital; IRB, 101, Daehak-ro, Jongno-gu, 03080, Seoul, KOREA, REPUBLIC OF	17-Jan-2014
265988	Schütte, Wolfgang	EK des Landes Sachsen-Anhalt, Kühnauer Str. 70, 06846, Dessau-Roßlau, GERMANY	20-Mar-2014
265989	von Pawel, Joachim	EK des Landes Sachsen-Anhalt, Kühnauer Str. 70, 06846, Dessau-Roßlau, GERMANY	20-Mar-2014

Site #	Investigator	IRB/IEC Name and Address	Approval Date
265990	Rittmeyer, Achim	EK des Landes Sachsen-Anhalt, Kühnauer Str. 70, 06846, Dessau-Roßlau, GERMANY	20-Mar-2014
265991	Schulz, Christian	EK des Landes Sachsen-Anhalt, Kühnauer Str. 70, 06846, Dessau-Roßlau, GERMANY	20-Mar-2014
265992	Grosch, Heidrun	EK des Landes Sachsen-Anhalt, Kühnauer Str. 70, 06846, Dessau-Roßlau, GERMANY	20-Mar-2014
265993	Engel-Riedel, Walburga	EK des Landes Sachsen-Anhalt, Kühnauer Str. 70, 06846, Dessau-Roßlau, GERMANY	24-Apr-2014
265994	KOLLMEIER, JENS	EK des Landes Sachsen-Anhalt, Kühnauer Str. 70, 06846, Dessau-Roßlau, GERMANY	20-Mar-2014
265995	SERKE, MONIKA	EK des Landes Sachsen-Anhalt, Kühnauer Str. 70, 06846, Dessau-Roßlau, GERMANY	20-Mar-2014
265996	Atmaca, Akin	EK des Landes Sachsen-Anhalt, Kühnauer Str. 70, 06846, Dessau-Roßlau, GERMANY	20-Mar-2014
266061	Lewanski, Conrad	Academic Health Science Centre; Joint Research Office, Imperial College London and Imperial College Healthcare NHS Trust, St Mary's Hospital, Mailbox 121, Praed Street, London, W2 1NY, UNITED KINGDOM	06-Aug-2014
266061	Lewanski, Conrad	London - Harrow Research Ethics Committee, Whitefriars, Lewins Mead, Level 3, Block B, Bristol, BS1 2NT, UNITED KINGDOM	06-Aug-2014
266064	SUMMERS, YVONNE	London - Harrow Research Ethics Committee, Whitefriars, Lewins Mead, Level 3, Block B, Bristol, BS1 2NT, UNITED KINGDOM	26-Sep-2014
266064	SUMMERS, YVONNE	The Christie NHS Foundation Trust; Research & Development Office, Block C, Withington Hall,Wilmslow Road, Withington, Manchester, M20 4BX, UNITED KINGDOM	26-Sep-2014
266068	Hennig, Ivo	Kings Mill Hospital; R&I Office, Sherwood Forest Hospitals NHS Foundation Trust, MANSFIELD ROAD, SUTTON IN ASHFIELD, SUTTON IN ASHFIELD, NG17 4JL, UNITED KINGDOM	20-Aug-2014
266068	Hennig, Ivo	London - Harrow Research Ethics Committee, Whitefriars, Lewins Mead, Level 3, Block B, Bristol, BS1 2NT, UNITED KINGDOM	20-Aug-2014
266069	BENEPAL, TIM	London - Harrow Research Ethics Committee, Whitefriars, Lewins Mead, Level 3, Block B, Bristol, BS1 2NT, UNITED KINGDOM	05-Nov-2014
266069	BENEPAL, TIM	St George's Hospital; St George’s Joint Research & Enterprise Office (JREO), Hunter Wing, Cranmer Terrace, London, SW17 0RE, UNITED KINGDOM	05-Nov-2014
266074	CHAO, DAVID	Royal Free Hampstead NHS Trust; Research and Developmemt Office, Admin Corridor Room G649, Medical School Building, Pond Street, London, NW3 2QG, UNITED KINGDOM	23-Oct-2014
266075	Upadhyay, Sunil	London - Harrow Research Ethics Committee, Whitefriars, Lewins Mead, Level 3, Block B, Bristol, BS1 2NT, UNITED KINGDOM	15-Oct-2014
266075	Upadhyay, Sunil	Northern Lincolnshire & Goole; Research and Development Department, Cliffe Gardens, Scunthorpe, DN15 7BH, UNITED KINGDOM	15-Oct-2014
266076	Spicer, James	Joint Clinical Trials Office; R&D., 16th Floor Tower Wing, Guy's Hospital, Great Maze Pond, London, SE1 9RT, UNITED KINGDOM	29-Sep-2014

Site #	Investigator	IRB/IEC Name and Address	Approval Date
266076	Spicer, James	London - Harrow Research Ethics Committee, Whitefriars, Lewins Mead, Level 3, Block B, Bristol, BS1 2NT, UNITED KINGDOM	29-Sep-2014
266163	YU, CHONG-JEN	Research Ethics Committee, Nat. Taiwan Univ. Hosp., No.1, Changde Street, Zhongzheng District, 100, TAIPEI, TAIWAN	27-Dec-2013
266164	KUO, HAN PIN	Chang Gung Med Found, Institutional Review Board, No. 123, Dunghu Rd., Jioulu Village, Taoyuan County, 333, Gueishan Township, TAIWAN	24-Feb-2014
266166	Hsia, Te-Chun	Research Ethics Committee China Medical University & Hospital, 2 Yude Road, 40447, Taichung, TAIWAN	04-Mar-2014
266169	CHEN, YUH-MIN	TVGH Institutional Review Board, No.201, Shih-Pai Road, Sec.2, 112, Taipei, TAIWAN	04-Mar-2014
266176	Forster, Martin	Joint Research Office, UCLH NHS Foundation Trust, 1st Floor Maple House (Suite B), 149 Tottenham Court Rd, London, W1T 7DN, UNITED KINGDOM	23-Oct-2014
266176	Forster, Martin	London - Harrow Research Ethics Committee, Whitefriars, Lewins Mead, Level 3, Block B, Bristol, BS1 2NT, UNITED KINGDOM	23-Oct-2014
266373	Galetta, Domenico	Comitato Etico Ospedale Oncologico di Bari	01-Apr-2014
266397	Acevedo Gaete, Alejandro Andres	Comité de Ética Servicio Salud Viña-Quillota; CEC Hospital Dr. Gustavo Fricke SSMV-Q, Av. Limache 1307, 2520563, Viña del Mar, CHILE	03-Jul-2014
266403	Aren, Osvaldo	Comite de Etica Servicio de Salud Metropolitano Norte, Maruri 272, Independencia, 8380656, Santiago, CHILE	10-Feb-2014
266593	Akewanlop, Charuwan	Ethics Committee, Faculty of Medicine, Siriraj Hospital, Mahidol University, 10700, Bangkok, THAILAND	10-Feb-2014
266596	SRIURANPONG, VIROTE	Institutional Review Board, Faculty of Medicine, Chulalongkorn University, 10330, Bangkok, THAILAND	18-Feb-2014
266598	Ativitavas, Touch	Ethical Clearance Committee on Human Rights, 270 RamaVI Road. Faculty of Medicine, Ramathibodi Hospital, Phayathai Rajathevi Bangkok 10400, 10400, Bangkok, THAILAND	22-Jan-2014
266618	Artal Cortes, Angel	Comité Ético de Investigación Clínica de Aragón, Avda Gómez Laguna 25, 50009, Zaragoza, ZARAGOZA, SPAIN	05-Feb-2014
266619	Garrido Lopez, Pilar	Hospital Ramon y Cajal ;Comité Etico de Investigación Clínica, Ctra. Colmenar Viejo, km 9,1, 28034, Madrid, MADRID, SPAIN	05-Feb-2014
266620	Ponce Aix, Santiago	CEIC Hospital Universitario 12 de Octubre, HOSPITAL MATERNO INFANTIL<br>Avda. de Córdoba, s/n, 28041, Madrid, MADRID, SPAIN	05-Feb-2014
266621	De Castro Carpeno, Javier	CEIC Área 5 - Hospital Universitario La Paz, Paseo de la Castellana, 261, 28036, Madrid, MADRID, SPAIN	05-Feb-2014
266622	Provencio Pulla, Mariano	Hospital Univ. Puerta de Hierro; Comité Etico de Investigacion Clinica, C/ SAN MARTÍN DE PORRES, 4, 28035, MADRID, MADRID, SPAIN	05-Feb-2014
266623	Cobo Dols, Manuel	CAEC-CEIC Autonomico de Ensayos Clinicos Andalucia, Avda. de la Innovación s/n, Edificio Arena 1, 41020, Sevilla, SEVILLA, SPAIN	05-Feb-2014
266624	Domine Gomez, Manuel	CEIC Fundacion Jimenez Diaz, Area Administrativa de Investigacion, Avd. Reyes Catolicos 2-2, 28040, Madrid, MADRID, SPAIN	05-Feb-2014
266625	Gonzalez Larriba, Jose Luis	CEIC Hospital Clinico San Carlos, Farmacologia/1 planta Norte. Puerta G., Professor Martin Lagos s/n, 28040, Madrid, MADRID, SPAIN	05-Feb-2014

Site #	Investigator	IRB/IEC Name and Address	Approval Date
266626	Rodriguez Abreu, Delvys	Comité Ético de Investigación Clínica, Hospital Insular de Las Palmas, Avda. Marítima del Sur, s/n, 35016, Las Palmas de Gran Canaria, LAS PALMAS, SPAIN	05-Feb-2014
266627	Garcia Campelo, M. Rosario	Comité Ético de Investigación Clínica de Galicia - Subdirección Xeral de Farmacia e Produtos Sanitar, Edificio Administrativo San Lázaro s/n, 15703, Santiago de Compostela - A Coruña, LA CORUÑA, SPAIN	05-Feb-2014
266685	Dichmann, Robert Andrew	Western Institutional Review Board WIRB Panel 7, 1019 39th Ave S.E., Puyallup, WA, 98374, UNITED STATES	23-Feb-2014
266686	DiCarlo, Brian Anthony	Western Institutional Review Board WIRB Panel 7, 1019 39th Ave S.E., Puyallup, WA, 98374, UNITED STATES	27-Feb-2014
266689	Emmons, Steven	Western Institutional Review Board WIRB Panel 7, 1019 39th Ave S.E., Puyallup, WA, 98374, UNITED STATES	23-Feb-2014
266690	Lawler, William Eyre	Western Institutional Review Board WIRB Panel 7, 1019 39th Ave S.E., Puyallup, WA, 98374, UNITED STATES	21-Feb-2014
266691	Lowe, Thomas Eugene	Western Institutional Review Board WIRB Panel 7, 1019 39th Ave S.E., Puyallup, WA, 98374, UNITED STATES	23-Feb-2014
266693	Sanchez, James Delfino	Western Institutional Review Board WIRB Panel 7, 1019 39th Ave S.E., Puyallup, WA, 98374, UNITED STATES	27-Feb-2014
266694	Tchekmedyian, Nerses Simon	Western Institutional Review Board WIRB Panel 7, 1019 39th Ave S.E., Puyallup, WA, 98374, UNITED STATES	27-Feb-2014
266696	Kim, David Dae-Young	Western Institutional Review Board WIRB Panel 7, 1019 39th Ave S.E., Puyallup, WA, 98374, UNITED STATES	24-Feb-2014
266716	BEARZ, ALESSANDRA	CEI IRCCS Centro di Rif. Oncologico di Aviano, VIA FRANCO GALLINI 2, 33081, AVIANO, Friuli-Venezia Giulia, ITALY	30-Apr-2014
266735	Baldini, Editta	Comitato Etico Regione Toscana - Area Vasta Nord Ovest , Via Roma 67, c/o Presidio Ospedaliero, 56126, Pisa, Toscana, ITALY	15-May-2014
266739	Johnson, Tirrell Tremayne	Western Institutional Review Board WIRB Panel 7, 1019 39th Ave S.E., Puyallup, WA, 98374, UNITED STATES	28-Feb-2014
266808	Pasello, Giulia	COMITATO ETICO DELL'ISTITUTO ONCOLOGICO VENETO, VIA GATTAMELATA, 64, 35128, Padova, Veneto, ITALY	14-Apr-2014
266846	SOTO PARRA, HECTOR	COMITATO ETICO CATANIA 1, VIA S. SOFIA 78, 95123, CATANIA, Sicilia, ITALY	02-Apr-2014
266847	Barlesi, Fabrice	CPP Sud-Méditerrannée I, Hopital Sainte Marguerite, 270 Boulevard Sainte Marguerite, 13274, Marseille, FRANCE	09-Apr-2014
266851	MONNET, ISABELLE	CPP Sud-Méditerrannée I, Hopital Sainte Marguerite, 270 Boulevard Sainte Marguerite, 13274, Marseille, FRANCE	09-Apr-2014
266852	DANSIN, ERIC	CPP Sud-Méditerrannée I, Hopital Sainte Marguerite, 270 Boulevard Sainte Marguerite, 13274, Marseille, FRANCE	09-Apr-2014
266853	DEBIEUVRE, DIDIER	CPP Sud-Méditerrannée I, Hopital Sainte Marguerite, 270 Boulevard Sainte Marguerite, 13274, Marseille, FRANCE	09-Apr-2014
266854	Decroisette Phan Van Ho, Chantal	CPP Sud-Méditerrannée I, Hopital Sainte Marguerite, 270 Boulevard Sainte Marguerite, 13274, Marseille, FRANCE	09-Apr-2014
266858	DENIS, FABRICE	CPP Sud-Méditerrannée I, Hopital Sainte Marguerite, 270 Boulevard Sainte Marguerite, 13274, Marseille, FRANCE	09-Apr-2014
266859	GERVAIS, RADJ	CPP Sud-Méditerrannée I, Hopital Sainte Marguerite, 270 Boulevard Sainte Marguerite, 13274, Marseille, FRANCE	09-Apr-2014
266860	GOLDWASSER, FRANCOIS	CPP Sud-Méditerrannée I, Hopital Sainte Marguerite, 270 Boulevard Sainte Marguerite, 13274, Marseille, FRANCE	09-Apr-2014
266862	Hilgers, Werner	CPP Sud-Méditerrannée I, Hopital Sainte Marguerite, 270 Boulevard Sainte Marguerite, 13274, Marseille, FRANCE	09-Apr-2014

Site #	Investigator	IRB/IEC Name and Address	Approval Date
266864	Corre, Romain	CPP Sud-Méditerrannée I, Hopital Sainte Marguerite, 270 Boulevard Sainte Marguerite, 13274, Marseille, FRANCE	09-Apr-2014
266867	Moro-Sibilot, Denis	CPP Sud-Méditerrannée I, Hopital Sainte Marguerite, 270 Boulevard Sainte Marguerite, 13274, Marseille, FRANCE	09-Apr-2014
266869	SCHOTT, ROLAND	CPP Sud-Méditerrannée I, Hopital Sainte Marguerite, 270 Boulevard Sainte Marguerite, 13274, Marseille, FRANCE	09-Apr-2014
266872	SOUQUET, PIERRE-JEAN	CPP Sud-Méditerrannée I, Hopital Sainte Marguerite, 270 Boulevard Sainte Marguerite, 13274, Marseille, FRANCE	09-Apr-2014
266873	TREDANIEL, JEAN	CPP Sud-Méditerrannée I, Hopital Sainte Marguerite, 270 Boulevard Sainte Marguerite, 13274, Marseille, FRANCE	09-Apr-2014
266874	WESTEEL, VIRGINIE	CPP Sud-Méditerrannée I, Hopital Sainte Marguerite, 270 Boulevard Sainte Marguerite, 13274, Marseille, FRANCE	09-Apr-2014
266887	POUESSEL, DAMIEN	CPP Sud-Méditerrannée I, Hopital Sainte Marguerite, 270 Boulevard Sainte Marguerite, 13274, Marseille, FRANCE	09-Apr-2014
266888	BERARD, HENRI	CPP Sud-Méditerrannée I, Hopital Sainte Marguerite, 270 Boulevard Sainte Marguerite, 13274, Marseille, FRANCE	09-Apr-2014
266889	CADRANEL, JACQUES	CPP Sud-Méditerrannée I, Hopital Sainte Marguerite, 270 Boulevard Sainte Marguerite, 13274, Marseille, FRANCE	09-Apr-2014
266890	MAZIERES, JULIEN	CPP Sud-Méditerrannée I, Hopital Sainte Marguerite, 270 Boulevard Sainte Marguerite, 13274, Marseille, FRANCE	09-Apr-2014
266984	FRIARD, SYLVIE	CPP Sud-Méditerrannée I, Hopital Sainte Marguerite, 270 Boulevard Sainte Marguerite, 13274, Marseille, FRANCE	09-Apr-2014
267102	SZCZESNA, ALEKSANDRA	Niezalezna Komisja Bioetyczna ds Badan Naukowych, Debinki 7, budynek nr 1, III pietro, 80-211, Gdansk, POLAND	27-Feb-2014
267103	Kowalski, Dariusz	Niezalezna Komisja Bioetyczna ds Badan Naukowych, Debinki 7, budynek nr 1, III pietro, 80-211, Gdansk, POLAND	27-Feb-2014
267105	Dziadziuszko, Rafal	Niezalezna Komisja Bioetyczna ds Badan Naukowych, Debinki 7, budynek nr 1, III pietro, 80-211, Gdansk, POLAND	27-Feb-2014
267106	Kalinka-Warzocha, Ewa	Niezalezna Komisja Bioetyczna ds Badan Naukowych, Debinki 7, budynek nr 1, III pietro, 80-211, Gdansk, POLAND	14-Apr-2014
267107	Losonczy, Gyorgy	Medical Research Council, Ethics Committee for Clinical Pharmacology, Arany J. u. 6-8., 1051, Budapest, HUNGARY	11-Apr-2014
267108	Soter, Szabolcs	Medical Research Council, Ethics Committee for Clinical Pharmacology, Arany J. u. 6-8., 1051, Budapest, HUNGARY	11-Apr-2014
267110	Sarosi, Veronika	Medical Research Council, Ethics Committee for Clinical Pharmacology, Arany J. u. 6-8., 1051, Budapest, HUNGARY	11-Apr-2014
267113	JOVANOVIC, DRAGANA	Ethics Committee Clinical Center Of Serbia, PASTEROVA 2, 11000, BELGRADE, SERBIA	18-Mar-2014
267114	Perin, Branislav	Ethics Committee Institute for Oncology of Vojvodina, Put doktora Goldmana 4, 21204, Sremska Kamenica, SERBIA	28-Feb-2014
267346	RAMLAU, RODRYG	Niezalezna Komisja Bioetyczna ds Badan Naukowych, Debinki 7, budynek nr 1, III pietro, 80-211, Gdansk, POLAND	14-Apr-2014
268160	Lantos, Ákos	Medical Research Council, Ethics Committee for Clinical Pharmacology, Arany J. u. 6-8., 1051, Budapest, HUNGARY	11-Apr-2014
268206	Yanez, Eduardo	Comité de Ética Cientifico del Servicio de Salud Araucania Sul, Vicuña Mackenna, 51, 4781086, Temuco, CHILE	14-May-2014
270244	Maemondo, Makoto	Miyagi Cancer Center Institutional Review Board, 47-1 Nodayama, Medeshima-Shiote, Natori, 981-1293, Miyagi, JAPAN	22-Apr-2014

Site #	Investigator	IRB/IEC Name and Address	Approval Date
270245	Horiike, Atsushi	The Cancer Institute Hospital of JFCR Institutional Review Board, 3-8-31 Ariake Koto-Ku, 135-8550, Tokyo, JAPAN	02-Apr-2014
270246	Nokihara, Hiroshi	National Cancer Center Institutional Review Board, 5-1-1 Tsukiji Chuo-Ku, 104-0045, Tokyo, JAPAN	11-Jun-2014
270247	Matsumoto, Shingo	National Cancer Center Institutional Review Board, 5-1-1 Tsukiji Chuo-Ku, 104-0045, Tokyo, JAPAN	13-Aug-2014
270249	Ikeda, Norihiko	Tokyo Medical University Hospital Institutional Review Board, 6-7-1 Nishishinjuku, Shinjuku-ku, 160-0023, Tokyo, JAPAN	13-May-2014
270250	Sakai, Hiroshi	Saitama Cancer Center Institutional Review Board, 780 Komuro Inamachi, Kitaadachi-gun, 362-0806, Saitama, JAPAN	16-Apr-2014
270251	TAKAHASHI, TOSHIAKI	Shizuoka Cancer Center Institutional Review Board, 1007 SHIMONAGAKUBO,NAGAIZUMI-CHO,SUNTOH-GUN, 411-8777, SHIZUOKA, JAPAN	17-Apr-2014
270252	HIDA, TOYOAKI	Aichi Cancer Center Hospital Institutional Review Board, 1-1 Kanokoden, Chikusaku, Nagoya-City, 464-8681, Aichi, JAPAN	24-Jun-2014
270253	NAKAGAWA, KAZUHIKO	Kindai University Hospital Institutional Review Board, 377-2 Ohnohigashi, Osaka-Sayama-shi, 589-8511, Osaka, JAPAN	21-Apr-2014
270254	Atagi, Shinji	National Hospital Organization Kinki-chuo Chest Medical Center Institutional Review Board, 1180 Nagasone-cho, Kita-ku, Sakai, 591-8555, Osaka, JAPAN	09-Apr-2014
270255	Satouchi, Miyako	Hyogo Cancer Center Institutional Review Board, 13-70 Kitaoujimachi,Akashi-shi, 673-8558, Hyogo, JAPAN	09-Apr-2014
270257	Kubo, Toshio	Okayama University Hospital Institutional Review Board, 2-5-1 Shikata-cho, Kita-ku, Okayama-city, 700-8558, Okayama, JAPAN	15-Apr-2014
270258	Chikamori, Kenichi	National Hospital Organization Yamaguchi-Ube Medical Center Institutional Review Board, 685 Higashikiwa Ube-shi, 755-0241, Yamaguchi, JAPAN	22-Apr-2014
270259	Kitajima, Hiromoto	National Hospital Organization Shikoku Cancer Center Institutional Review Board, 160 Minamiumemotomachi-Kou, Matsuyama-shi, 791-0280, Ehime, JAPAN	19-May-2014
270261	Seto, Takashi	National Hospital Organization Kyushu Cancer Center; IRB, 3-1-1 Notame, Minami-ku, Fukuoka-shi, 811-1395, Fukuoka, JAPAN	07-May-2014
270783	Fujita, Shiro	Institute of Biomedical Research and Innovation Institutional Review Board, 2-2 Minatojimaminamimachi Chuo-ku Kobe-city, 650-0047, Hyogo, JAPAN	23-Apr-2014
272323	FEHRENBACHER, LOUIS	Kaiser Permanente Northern California IRB, 1800 Harrison Street, 16th Floor, Oakland, CA, 94612, UNITED STATES	18-Mar-2014
272324	FEHRENBACHER, LOUIS	Kaiser Permanente Northern California IRB, 1800 Harrison Street, 16th Floor, Oakland, CA, 94612, UNITED STATES	18-Mar-2014
272325	FEHRENBACHER, LOUIS	Kaiser Permanente Northern California IRB, 1800 Harrison Street, 16th Floor, Oakland, CA, 94612, UNITED STATES	18-Mar-2014
272326	FEHRENBACHER, LOUIS	Kaiser Permanente Northern California IRB, 1800 Harrison Street, 16th Floor, Oakland, CA, 94612, UNITED STATES	18-Mar-2014
272328	FEHRENBACHER, LOUIS	Kaiser Permanente Northern California IRB, 1800 Harrison Street, 16th Floor, Oakland, CA, 94612, UNITED STATES	18-Mar-2014
272329	FEHRENBACHER, LOUIS	Kaiser Permanente Northern California IRB, 1800 Harrison Street, 16th Floor, Oakland, CA, 94612, UNITED STATES	08-Mar-2014
272330	FEHRENBACHER, LOUIS	Kaiser Permanente Northern California IRB, 1800 Harrison Street, 16th Floor, Oakland, CA, 94612, UNITED STATES	18-Mar-2014

Site #	Investigator	IRB/IEC Name and Address	Approval Date
272331	FEHRENBACHER, LOUIS	Kaiser Permanente Northern California IRB, 1800 Harrison Street, 16th Floor, Oakland, CA, 94612, UNITED STATES	18-Mar-2014
272696	Chella, Antonio	Comitato Etico Regione Toscana - Area Vasta Nord Ovest , Via Roma 67, c/o Presidio Ospedaliero, 56126, Pisa, Toscana, ITALY	10-Jul-2014
